# Development of New Resolvin D1 Analogues for Osteoarthritis Therapy: Acellular and Computational Approaches to Study Their Antioxidant Activities

**DOI:** 10.3390/antiox13040386

**Published:** 2024-03-22

**Authors:** Zahra Kariminezhad, Mahdi Rahimi, Julio Fernandes, René Maltais, Jean-Yves Sancéau, Donald Poirier, Hassan Fahmi, Mohamed Benderdour

**Affiliations:** 1 Orthopedic Research Laboratory, Hôpital du Sacré-Cœur de Montréal, Université de Montréal, Montréal, QC H4J 1C5, Canada; zahra.kariminezhad.cnmtl@ssss.gouv.qc.ca (Z.K.); mahdi.rahimi.hoseinabad@umontreal.ca (M.R.); julio.c.fernandes@umontreal.ca (J.F.); 2 Department of Molecular Medicine, Faculty of Medicine, Université Laval, Québec, QC G1V 0A6, Canada; rene.maltais@crchudequebec.ulaval.ca (R.M.); jean-yves.sanceau@crchudequebec.ulaval.ca (J.-Y.S.); donald.poirier@crchudequebec.ulaval.ca (D.P.); 3 Organic Synthesis Service, Medicinal Chemistry Platform, CHU de Québec Research Center, Université Laval, Québec, QC G1V 4G2, Canada; 4 Osteoarthritis Research Unit, University of Montreal Hospital Research Center (CRCHUM), Montréal, QC H2X 0A9, Canada; h.fahmi@umontreal.ca

**Keywords:** Resolvin D1, analogues, osteoarthritis, antioxidant, reactive oxygen species, hyaluronic acid

## Abstract

In osteoarthritis (OA), oxidative stress plays a crucial role in maintaining and sustaining cartilage degradation. Current OA management requires a combination of pharmaceutical and non-pharmacological strategies, including intraarticular injections of hyaluronic acid (HA). However, several lines of evidence reported that HA oxidation by reactive oxygen species (ROS) is linked with HA cleavage and fragmentation, resulting in reduced HA viscosity. Resolvin D1 (RvD1) is a lipid mediator that is biosynthesized from omega-3 polyunsaturated fatty acids and is a good candidate with the potential to regulate a panoply of biological processes, including tissue repair, inflammation, oxidative stress, and cell death in OA. Herein, newly designed and synthesized imidazole-derived RvD1 analogues were introduced to compare their potential antioxidant properties with commercially available RvD1. Their antioxidant capacities were investigated by several in vitro chemical assays including oxygen radical absorbance capacity, 2,2-diphenyl-1-picrylhydrazyl radical scavenging, ferric ion reducing antioxidant power, hydroxyl radical scavenging, and HA fragmentation assay. All results proved that imidazole-derived RvD1 analogues showed excellent antioxidant performance compared to RvD1 due to their structural modifications. Interestingly, they scavenged the formed reactive oxygen species (ROS) and protected HA from degradation, as verified by agarose gel electrophoresis and gel permission chromatography. A computational study using Gaussian 09 with DFT calculations and a B3LYP/6-31 G (d, p) basis set was also employed to study the relationship between the antioxidant properties and chemical structures as well as calculation of the molecular structures, frontier orbital energy, molecular electrostatic potential, and bond length. The results showed that the antioxidant activity of our analogues was higher than that of RvD1. In conclusion, the findings suggest that imidazole-derived RvD1 analogues can be good candidates as antioxidant molecules for the treatment of oxidative stress-related diseases like OA. Therefore, they can prolong the longevity of HA in the knee and thus may improve the mobility of the articulation.

## 1. Introduction

**Figure 1:**
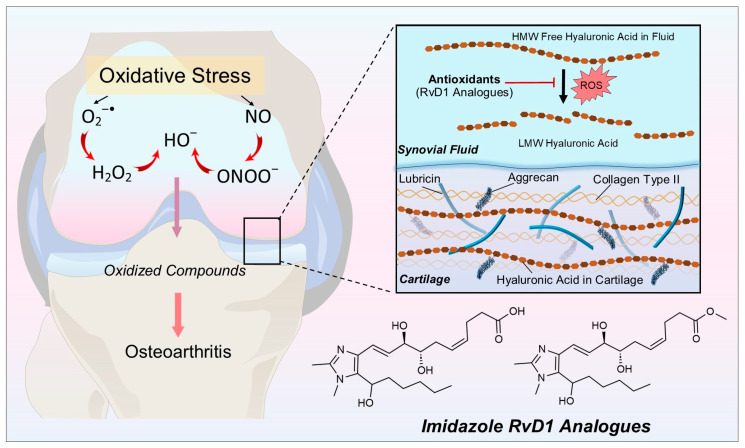
Oxidative stress and its mechanism in osteoarthritis: the primarily formed radicals, superoxide radical (O^2−•^), can be converted to more harmful species such as hydroxyl radicals (OH^•^) and hydrogen peroxide (H_2_O_2_). Imidazole-derived RvD1 analogues as antioxidant molecules scavenge ROS via their active sites to protect against the degradation of high molecular weight hyaluronic acid (HMW HA) to low molecular weight hyaluronic acid (LMW HA).

Osteoarthritis (OA) is known as a chronic degenerative and musculoskeletal disorder that causes pain and physical disability in patients’ quality of life [1,2]. Although OA is considered a global disease affecting all joint tissues (subchondral bone, ligaments, capsule, synovial membrane, and menisci), cartilage degradation is the end point of this disease [3]. The degradation of cartilage can be started by the combination of mechanical stress and biochemical factors. One of the most important biochemical alterations in OA is oxidative stress, which is characterized by the increased formation of reactive oxygen species (ROS), reactive nitrogen species (RNS), lipid peroxidation (LPO) products, and the insufficiency of biological molecules to scavenge these hazardous species [4,5,6,7]. ROS/RNS can be classified as either free radicals (containing one unpaired electron) or non-radicals. The radicals molecules include superoxide radical (O2^•−^), hydroxyl radical (HO^●^), nitric oxide radical (^●^NO), peroxyl (ROO^●^), and alkoxyl radicals (RO^●^) [8]. In contrast, the non-radical contains hydrogen peroxide (H_2_O_2_), organic peroxide (ROOR′), ozone (O_3_), hypochlorous acid (HClO), singlet oxygen (^1^O_2_), aldehydes (HCOR), peroxynitrite (ONOOH), etc. [8,9]. At controlled levels, ROS act as signaling molecules in cells. However, an imbalance between ROS and antioxidants, known as redox state dysregulation, disrupts cell function and can contribute to disease. In OA, ROS and LPO accumulation cause cartilage damage by increasing the degradation of the extracellular matrix (ECM) and decreasing the synthesis of proteoglycan and type II collagen [7,10,11,12]. Hyaluronic acid (HA), a naturally occurring non-sulfated glycosaminoglycane (GAG), is found in synovial fluid, cell surface, and ECM tissue. It is composed of disaccharides containing glucuronic acid and N-acetylglucosamine which is synthesized by the HA synthase (HAS) [13]. HA is initially present as a polymer with high molecular weight (HMW), but it undergoes degradation by hyaluronidase (HYAL) or ROS/RNS actions, leading to it breaking down into smaller fragments and oligosaccharides with low molecular weight (LMW) [14]. HMW HA has anti-inflammatory, immunosuppressive, and antiangiogenic effects, whereas LMW HA has pro-inflammatory, pro-angiogenetic, and oncogenic effects [15]. ROS/RNS naturally degrades HMW HA albeit at enhanced levels during tissue injury and inflammatory processes [16,17]. Several HA formulations were developed to protect its oxidation and degradation. These formulations include the combination of HA with sorbitol and mannitol. These compounds can prolong the longevity of injected HA in the knee and thus may improve the mobility and flexibility of the articulation [18,19,20,21]. At the cellular level, these formulations restore redox status and reduce markers of apoptosis, inflammation and catabolism involved in cartilage damage [22,23]. Thus, the degradation of HA by increased ROS challenges vascular integrity and can initiate OA disease progressions (Figure 1).

Growing evidence implicates ROS pathways in the development of OA. Leveraging this knowledge, researchers are exploring innovative antioxidant-based interventions. These interventions aim to reduce harmful oxidative stress and bolster cellular defenses, potentially paving the way for innovative OA management strategies [24]. Enzymatic and non-enzymatic antioxidants are responsible for controlling and inhibiting oxidative enzymes, scavenging free radicals, or chelating ion metals [25]. There are two complementary antioxidant defense systems against oxidative stress: the enzymatic system (SOD, GPX, CAT) catalyzes ROS conversion, while the non-enzymatic system (GSH, vitamin C, vitamin E, carotenoids) directly quenches ROS or participates in redox cycles [26]. Antioxidants, derived from both endogenous and exogenous sources, exert crucial functions in free radical scavenging, prevention, and the neutralization of pre-radical molecules. The development of antioxidant-based supplements and drugs offers a potential avenue for reinforcing cellular antioxidant status and mitigating the detrimental effects of oxidative stress. Resolvins are a class of compounds derived from omega-3 fatty acids and subclassed in three categories including Resolvin Ds, Resolvin Es, Resolvin Ts, Resolvin-3DPA, and Resolvin-6DPA [27,28]. Among these, Resolvin D1 (RvD1) has been suggested as a novel treatment option for several inflammatory diseases due to its remarkable properties in resolving inflammation, promoting tissue repair, preserving tissue integrity, and the treatment of bone and cartilage disorders [29,30,31,32,33,34]. However, RvD1 is a chemically unstable molecule that undergoes a very fast transformation to inactive products through the enzymatic action of eicosanoid oxidoreductase [35,36]. To improve its metabolic stability, research has been studied to develop and synthesize metabolically stable analogues with improved pharmacokinetic and pharmacodynamic properties.

Very recently, our research team designed and synthesized a series of RvD1 analogues and conducted several biological studies to present their abilities to inhibit inflammatory processes and antioxidant properties [37]. Herein, we performed in vitro assays to measure the antioxidant capacity of newly designed imidazole-derived RvD1 analogues and simulated their activity by computational study. In this regard, oxygen radical absorbance capacity (ORAC) and hydroxyl radical scavenging with copper II (HRS), 2,2-diphenyl-1-picrylhydrazyl (DPPH), and ferric reducing antioxidant power (FRAP) assays were performed to measure the total antioxidant status and determine the antioxidant activity of the analogues. The effectiveness of imidazole-derived RvD1 analogues in scavenging radicals was evaluated by incubating HMW HA, an important component of cartilage and synovial fluid, with ROS. The protection of HMW HA from degradation was checked by agarose gel electrophoresis and gel permeation chromatography. Moreover, the quantum chemical simulation was used to characterize the molecular structure, and the density functional theory (DFT) study provided useful data related to antioxidant capacity such as O-H bond length, chemical reactivity, and the energy of frontier orbitals. A DFT study was also performed to analyze the antioxidant activity and the active sites of RvD1 and its analogues.

## 2. Materials and Methods

### 2.1. Materials

Chemical reagents including fluorescein sodium salt (NaFluo), 2,2′-azobis(2-methylpropionamidine) dihydrochloride (AAPH, granules, 97%), and 2,2-diphenyl-1-picrylhydrazyl (DPPH, powder) were purchased from MilliporeSigma Co. (St. Louis, MO, USA). The antioxidants as controls including ascorbic acid (AA, 99%) and (±)-6-hydroxy-2,5,7,8-tetramethylchromane-2-carboxylic acid (Trolox, 97%) were also obtained from MilliporeSigma Co. (St. Louis, MO, USA). A Ferric Reducing Antioxidant Power (FRAP) Assay Kit (Colorimetric) was purchased from Invitrogen by Thermo Fisher Scientific (Frederick, MD, USA). Resolvin D1 was purchased from Cayman Chemical Co. (Ann Arbor, MI, USA) and stored at −80 °C. Two imidazole-derived Resolvin D1 analogues including (4Z,7R,8R,9E)-7,8-dihydroxy-10-(5-(1-hydroxyhexyl)-1,2-dimethyl-1H-imidazol-4-yl)deca-4,9-dienoic acid (Analogue 1) and methyl (4Z,7S,8R,9E)-7,8-dihydroxy-10-(5-(1-hydroxyhexyl)-1,2-dimethyl-1H-imidazol-4-yl)deca-4,9-dienoate (Analogue 2) were synthesized by Poirier’s research group. The purity of the final compound’s RvD1 analogues 1 and 2 to be tested was determined with a Shimadzu HPLC (Tokyo, Japan) apparatus equipped with an SPD-M20A photodiode array detector, a Setima HPLC18 reversed-phase column (250 mm × 4.6 mm) and using a solvent gradient of MeOH:water from 70:30 to 100:0 on a 30 min run. The wavelength of the UV detector was 245 nm.

### 2.2. Analogues Synthesis

#### 2.2.1. **Block A** Synthesis

The **block A** synthesis sequence was adapted from de Gaetano et al. [38].

(*i*) 2-Deoxy-3,4-*O*-(1-methylethylidene)-D-*erythro*-pentopyranose (**1**)

To a solution of 2-desoxy-D-ribose (18.7 mmol, 2.5 g) in anhydrous DMF (25 mL) under an argon atmosphere was added drierite dessicant (1.0 g), dimethoxy-propane (37 mmol, 3.8 g, 4.5 mL) and p-toluenesulfonic (p-TSA) (0.55 mmol, 0.1 g). The solution was stirred for 3 h at room temperature. Triethylamine (TEA) (0.1 mL) was then added before the mixture was filtered over a celite pad. The filtrate was evaporated under pressure. The crude compound was purified by flash chromatography with EtOAc/hexanes (7:3 + 1% TEA) to give 1.7 g (53%) of compound **1**. The ^1^H NMR spectra was in full agreement with the literature data.

(*ii*) (Methoxycarbonyl propyl) triphenylphosphonium bromide (**2**)

To a solution of methyl-4-bromo-butanoate (25.0 g, 140 mmol) in toluene (250 mL) was added triphenylphosphine (33.5 g, 170 mmol), after which it was stirred at 100 °C for 24 h. The resulting solid was then filtered and washed with cold toluene to give compound **2** as a white solid (12.0 g, 20%).

(*iii*) Methyl (4*Z*)-6-[(4*S*,5*R*)-5-(hydroxymethyl)-2,2-dimethyl-1,3-dioxolan-4-yl] hex-4-enoate (**3**)

To a solution of phosphonium salt **2** (9.6 g, 21 mmol) in anhydrous tetrahydrofuran (THF) (60 mL) at −78 °C under an argon atmosphere was dropwise added potassium hexamethyldisilazide (KHMDS) (1.0 M in THF, 20 mmol, 20.0 mL) over 10 min. The solution was then allowed to return at 0 °C and was stirred for 1 h. After the solution cooled down to −78 °C, compound **1** (1.5 g, 8.7 mmol) was added, and the solution was stirred for 20 min at this temperature before being warmed to room temperature and stirred for an additional 30 min. The resulting solution was poured into a saturated ammonium chloride solution and extracted with EtOAc. The organic layer was washed with brine, dried over sodium sulfate, filtered, and evaporated under reduced pressure. The resulting crude compound was purified by flash chromatography with EtOAc/hexanes (1:1 + 1% TEA) to give 0.67 g (32%) of compound **3**. The ^1^H NMR spectra was in full agreement with the literature data.

(*iv*) Methyl (4*Z*)-6-[(4*S*,5*S*)-5-formyl-2,2-dimethyl-1,3-dioxolan-4-yl] hex-4-enoate (**4**)

To a solution of compound **3** (2.8 mmol, 0.67 g) in dichloromethane (DCM) (10 mL) was dropwise added a solution of Dess–Martin periodinane (5.6 mol, 2.4 g) in 10 mL of DCM. The solution was stirred for 2 h and then poured into a saturated solution of sodium bisulfite. The solution was then extracted with EtOAc. The organic layer was washed with a saturated solution of sodium bicarbonate, and then with brine, it was dried with sodium sulfate, filtered and evaporated under reduced pressure. The resulting crude product **4** was used for the next step. The ^1^H NMR spectra was in full agreement with the literature data.

(*v*) Methyl (4*Z*)-6-[(4*S*,5*R*)-5-ethynyl-2,2-dimethyl-1,3-dioxolan-4-yl] hex-4-enoate (**5**)

To K_2_CO_3_ (7.9 mmol, 1.1 g) under an argon atmosphere was dropwise added a solution of phosphonate (3.9 mmol, 0.67 g) in MeOH (10 mL). To the later solution was dropwise added a solution of aldehyde **4** (2.8 mmol, 0.67 g) in MeOH (45 mL) at room temperature, and the solution was stirred for 3 h at room temperature. The resulting solution was poured into water, extracted with EtOAc, washed with brine, dried over sodium sulfate, filtered and evaporated under reduced pressure. The crude compound was purified by flash chromatography with EtOAc/hexanes (3:7 + 1% TEA) to give 330 mg (50%) of compound **5**. ^1^H NMR (400 MHz, Acetone-*d*_6_) 5.57–5.36 (m, 2H), 4.77 (dd, *J* = 5.5, 2.2 Hz, 1H), 4.10 (ddd, *J* = 7.2, 6.5, 5.5 Hz, 1H), 3.59 (s, 3H), 3.05 (d, *J* = 2.2 Hz, 1H), 2.56–2.42 (m, 2H), 2.42–2.26 (m, 2H), 1.43 (s, 3H), 1.26 (s, 3H).^13^C NMR (100.6 MHz, Acetone-d_6_) 172.7, 130.1, 125.8, 110.0, 109.3, 77.4, 76.3, 68.8, 50.7, 33.3, 29.4, 27.2, 25.6, 22.8. LRMS (APCI pos) *m*/*z* 253.1 [M + H].

(*vi*) Methyl (4*Z*)-6-{(4*S*,5*R*)-2,2-dimethyl-5-[(*E*)-2-(4,4,5,5-tetramethyl-1,3,2-dioxaborolan-2-yl)ethenyl]-1,3-dioxolan-4-yl}hex-4-enoate (**Block-A**)

In a microwave vial under an argon atmosphere was added compound **5** (1.3 mmol, 330 mg), TEA (0.4 mmol, 0.04 mL) and pinacolborane (2.5 mmol, 0.38 mL). To this mixture was added the Schwartz reagent (0.13 mmol, 34 mg), and the solution was heated at 60 °C for 36 h. The resulting solution was poured into water, extracted with EtOAc, washed with brine, dried over sodium sulfate, filtered, and evaporated under reduced pressure. The crude compound was purified by flash chromatography with EtOAc/hexanes (2:8 + 1% TEA) to give 105 mg (21%) of **Block-A**. ^1^H NMR (400 MHz, Acetone-*d*_6_) 6.60–6.39 (m, 1H), 5.64 (dd, *J* = 18.0, 1.4 Hz, 1H), 5.44–5.33 (m, 2H), 4.58 (td, *J* = 6.4, 1.4 Hz, 1H), 4.23 (dt, *J* = 7.7, 6.3 Hz, 1H), 3.58 (s, 3H), 2.41–2.24 (m, 4H), 2.18 (td, *J* = 7.4, 6.7, 4.5 Hz, 2H), 1.41 (d, *J* = 0.7 Hz, 3H), 1.28 (d, *J* = 0.7 Hz, 3H), 1.22 (s, 12H). ^13^C NMR (100.6 MHz, Acetone-d_6_) 172.6, 148.8, 129.6, 126.5, 108.0, 83.0, 79.8, 77.9, 50.7, 33.3, 27.5, 24.8, 24.2, 22.9. LRMS (APCI pos) *m*/*z* 381.2 [M + H].

#### 2.2.2. **Block B** Synthesis

**Figure 2:**
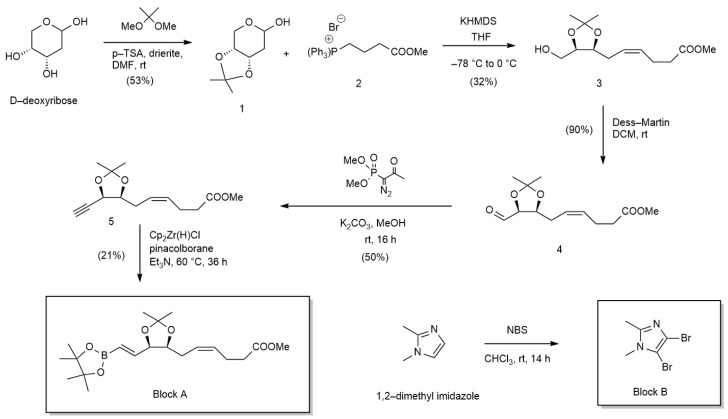
Chemical synthesis of **Block A** and **Block B**. Reagents, conditions, and reaction yields.

**Block B** was prepared following a literature procedure [38], as shown in Figure 2. Briefly, 1,2-dimethylimidazole was treated with N-bromosuccinimide (2.2 eq) in chloroform and reacted for 14 h at room temperature to give the dibromoimidazole compound—**Block B.** The reaction was quenched, and the compound was isolated and purified. The ^1^H NMR data were in full agreement with that reported in the literature.

#### 2.2.3. RvD1 Analogues 1 and 2 Syntheses

(*i*) 1-(4-Bromo-1,2-dimethyl-1H-imidazol-5-yl) hexan-1-one (**6**)

**Figure 3:**
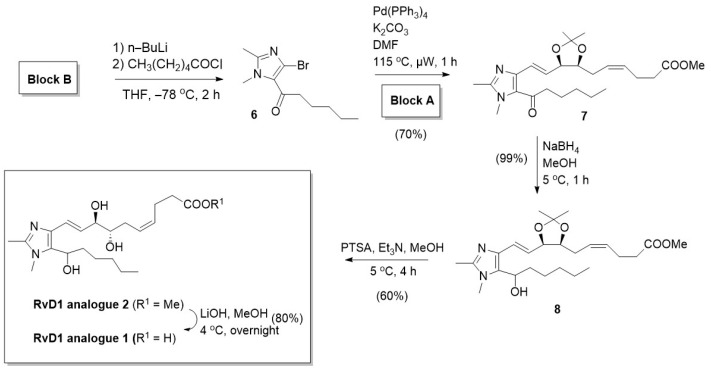
Chemical synthesis of **RvD1 analogues 1** and **2**. Reagents, conditions and reaction yields.

This compound was prepared following a literature procedure [38] (Figure 3). The ^1^H NMR data were in full agreement with that reported in the literature.

(*ii*) Methyl (4*Z*)-6-{(4*S*,5*R*)-5-[(*E*)-2-(5-hexanoyl-1,2-dimethyl-1H-imidazol-4-yl) ethenyl]-2,2-dimethyl-1,3-dioxolan-4-yl} hex-4-enoate (**7**)

In a 20 mL microwave vial, compound **6** (54 mg, 0.20 mmol) and **Block A** (100 mg, 0.26 mmol) were solubilized in dimethylformamide (DMF) (1.2 mL) and 2M K_2_CO_3_ (0.2 mL). Argon was bubbled through the mixture for 15 min before the addition of Pd (PPh_3_)_4_ (20 mg, 0.016 mmol). After sealing, the tube was heated in a microwave apparatus at 115 °C for 1 h. After cooling at room temperature, water was added, and the aqueous phase was extracted with EtOAc. The combined extracts were washed with water, brine and dried over Na_2_SO_4_. The residue was purified on silica gel eluting with toluene–EtOAc–ether (90:10:10 to 70:30:10) affording compound **7** (61 mg, 70%). ^1^H NMR shows contamination with triphenylphosphine oxide and (1,2-dimethyl-1H-imidazol-5-yl) hexan-1-one. ^1^H NMR (400 MHz, Methanol-*d*_4_) 6.86 (d, *J* = 15.3 Hz, 1H), 6.43 (dd, *J* = 15.3, 6.8 Hz, 1H), 5.33 (m, 2H), 4.68 (m, 1H), 4.21 (q, *J* = 6.6 Hz, 1H), 3.65 (s, 3H), 3.49 (s, 3H), 2.29 (s, 3H), 2.18 (s, 6H), 1.58 (m, 2H), 1.41 (s, 3H), 1.26 (m, 7H), 0.81 (bs, 3H).

(*iii*) Methyl (4*Z*)-6-[(4*S*,5*R*)-5-{(*E*)-2-[5-(1-hydroxyhexyl)-1,2-dimethyl-1H-imidazol-4-yl]ethenyl}-2,2-dimethyl-1,3-dioxolan-4-yl]hex-4-enoate (**8**)

To an ice-cooled solution of compound **7** (60 mg, 0.13 mmol) in MeOH (1 mL) was added NaBH_4_ (10 mg, 0.26 mmol). The mixture was stirred at 5 °C for 1 h and then quenched with a 10% aqueous solution of NaH_2_PO_4_. The aqueous phase was extracted twice with EtOAc. The combined extracts were washed with water, brine and dried over Na_2_SO_4_. Evaporation gave quantitatively compound **8** (60 mg). ^1^H NMR (400 MHz, Methanol-*d*_4_) 6.59 (d, *J* = 15.6 Hz, 1H), 6.21 (dd, *J* = 15.6, 8.1 Hz, 1H), 5.43 (m, 2H), 4.68 (m, 1H), 4.21 (m, 1H), 3.65 (s, 3H), 3.62 (s, 3H), 2.30 (s, 3H), 2.18 (s, 3H), 1.92 (m, 1H), 1.76 (m, 1H), 1.49–1.19 (m, 6H), 0.88 (bs, 3H).

(*iv*) Methyl (4*Z*,7*S*,8*R*,9*E*)-7,8-dihydroxy-10-[5-(1-hydroxyhexyl)-1,2-dimethyl-1H-imidazol-4-yl]deca-4,9-dienoate (**RvD1 analogue 2**)

To a solution of crude compound **8** (60 mg, 0.13 mmol) in MeOH (1 mL) was added p-TSA (39 mg, 0.21 mmol) at 5 °C, after which it was stirred for 4 h. TEA was added, and MeOH was evaporated under reduced pressure. The residue was poured into water and extracted with EtOAc. The combined extracts were washed with water, brine, dried over sodium sulfate (Na_2_SO_4_), filtered, and evaporated under reduced pressure to give crude material. This later compound was purified by flash chromatography using DCM/MeOH (9:1) as eluent to give compound **RvD1 analogue 2** (33 mg, 60%). ^1^H NMR (400 MHz, Methanol-*d*_4_) δ 6.58 (d, *J* = 15.7 Hz, 1H), 6.27 (dd, *J* = 15.1, 7.3 Hz, 1H), 5.55 (m, 2H), 5.45 (m, 1H), 4.58 (m, 1H), 4.08 (m, 1H), 3.64 (s, 3H), 3.59 (s, 3H), 2.37 (m, 3H), 1.93–1.71 (m, 2H), 1.49–1.17 (m, 6H), 0.88 (bs, 3H). ^13^C NMR (100.6 MHz, Methanol-*d*_4_) 174.0, 146.0, 149.9, 133.5, 130.1, 128.9, 127.4, 127.2, 126.6, 126.5, 121.6, 75.5, 75.4, 74.6, 74.5, 64.9, 50.6, 36.1, 36.0, 33.4, 33.3, 31.4, 31.3, 30.7, 30.2, 25.5, 25.4, 22.6, 22.3, 12.9, 11.2. LRMS (APCI pos) *m*/*z* 409.3 [M + H]. HPLC purity = 99.6%.

(*v*) (4*Z*,7*S*,8*R*,9*E*)-7,8-dihydroxy-10-[5-(1-hydroxyhexyl)-1,2-dimethyl-1H-imidazol-4-yl] deca-4,9-dienoic acid (**RvD1 analogue 1**)

To a solution of compound **RvD1 analogue 2** (22 mg, 0.06 mmol) in MeOH (0.5 mL) was added LiOH (16 mg, 0.38 mmol). The mixture was stirred at 4 °C overnight and then poured into an aqueous solution (10%) of NaH_2_PO_4_. The aqueous solution was extracted with EtOAc, washed with brine, dried over Na_2_SO_4_, filtered, and evaporated under reduced pressure to give 17 mg (80%) of compound **RvD1 analogue 1** as a yellow solid. ^1^H NMR (400 MHz, Methanol-*d*_4_) δ 6.65 (d, *J* = 15.7 Hz, 1H), 6.42 (dd, *J* = 15.1, 7.3 Hz, 1H), 5.51 (m, 2H), 4.94 (m, 1H), 4.18 (m, 1H), 3.75 (s, 3H), 3.65 (m, 1H), 2.51 (s, 3H), 2.47–2.38 (m, 2H), 2.38–2.23 (m, 4H), 1.93–1.77 (m, 2H), 1.49–1.19 (m, 6H), 0.90 (bs, 3H). ^13^C NMR (100.6 MHz, Methanol-*d*_4_) 177.2, 145.6, 145.5, 130.9, 130.8, 130.2, 130.1, 129.9, 129.3, 126.4, 126.1, 117.5, 117.4, 74.43, 74.41, 74.4, 74.3, 64.7, 64.6, 35.96, 35.9, 34.8, 31.33, 31.32, 31.3, 30.4, 30.3, 29.3, 25.34, 25.33, 23.3, 22.2, 12.9, 10.1, 10.0. LRMS (APCI pos) *m*/*z* 395.3 [M + H]. HPLC purity = 100.0%.

All the results of the analogues’ synthesis, including the ^1^H NMR spectrum, ^13^C NMR spectrum, LCMS chromatogram, and mass spectrum, are described in Figures S1–S9.

### 2.3. Antioxidant Activity Assays

#### 2.3.1. Oxygen Radical Absorbance Capacity (ORAC) Assay

The ORAC assay was performed as described by Huang et al. with the following modifications [39]. Briefly, different concentrations of antioxidant samples were prepared in phosphate buffer solution (PBS 75 mM, pH 7.4). Then, a fresh stock solution of fluorescein (58 nM) in PBS was prepared in a separate conical tube and stored at 4 °C. Afterward, 20 µL of different concentrations of antioxidants was mixed with 120 µL of fluorescein probe in a 96-well plate and incubated at 37 °C for 30 min. The fluorescence intensity (excitation 485 nm, emission 520 nm) was measured to determine the background signal by fluorometer. Subsequently, 60 µL of 2,2′-azobis(2-methylpropionamidine) dihydrochloride (AAPH) (40 mM) was added manually with a multichannel pipette for all wells except for the negative control (fluorescein probe without AAPH). The plate was scanned for 250 cycles every 60 s. The final concentrations of the antioxidants in the wells are as follows: 20, 10, 5, 2.5, 1.25, and 0.625 µM. The area under the curve (AUC) and the Net AUC for all samples and standards were measured using Equations (1) and (2), respectively.

AUC = (R_1_/R_1_) + (R_2_/R_1_) + (R_3_/R_1_) + ⋯ + (R_n_/R_1_) (1)

R_1_ = relative fluorescence value of time points zero.

R_n_ = relative fluorescence value of time points (e.g., R_3_ is relative fluorescence value at minute = 3).

NET AUC = AUC _Antioxidant_ − AUC _Blank_ (2)

The standard curve was obtained by plotting the Net AUC against the concentrations of different antioxidant samples. For comparison of the data, the antioxidant activity of all samples was converted into Trolox equivalent (TE) according to Equation (3).

TE = Slope _Sample_/Slope _Trolox_ (3)

#### 2.3.2. DPPH Assay

The 2,2-diphenyl-1-picrylhydrazyl (DPPH) method was performed according to the method discovered by Goldschmidt and Renn in the 1920s and then developed by Blois in 1958 [40,41]. Firstly, different concentrations of antioxidants were prepared in deionized water. Then, in a separate conical tube, a fresh stock solution of DPPH reagent (600 µM) was prepared in methanol and stored at 4 °C. In a 96-well plate, 50 µL of different concentrations of antioxidants was mixed gently with 50 µL of DPPH solution and incubated in a dark condition at room temperature for 30 min. The well containing DPPH without antioxidants is considered as a negative control, and the final concentrations of the antioxidants in the wells are as follows: 20, 10, 5, 2.5, 1.25, and 0.625 µM. The absorbance was measured by an ELISA microplate reader at 517 nm. The scavenging percentage of all samples was measured using Equation (4).

DPPH Scavenging% = [(Abs _control_ − Abs _sample_)/(Abs _control_)] × 100 (4)

#### 2.3.3. FRAP Assay

The ferric reducing antioxidant power (FRAP) assay was developed by Iris Benzie and J.J. Strain [42]. To determine the antioxidant activity of the samples, the experiment procedure was completed according to the protocol provided by the manufacturer. In a 96-well plate, 25 µL of standards or different concentrations of samples was mixed with 75 µL FRAP color solution and then incubated at room temperature for 30 min. The final concentrations of the antioxidants in the wells were as follows; 20, 10, 5, 2.5, 1.25, 0.625, and 0.3125 µM. Subsequently, the absorbance was read at 593 nm by UV spectrophotometer. According to the FRAP kit protocol, a serial dilution of ferrous chloride (FeCl_2_) standard was prepared in assay buffer 1X (provided by FRAP kit) to plot the calibration curve. The relative percentage of reducing power in compared Trolox for all antioxidants samples was calculated according to the following formula [43]:

Relative % of Reducing Power = (Abs _samaple_ − Abs _min_)/Abs _max_ − Abs _min_) × 100 (5)

where Abs—absorbance of sample, Abs _min_—absorbance of control, and Abs _max_—highest Abs of control (Trolox).

#### 2.3.4. Hydroxyl Radical Scavenging (HRS)

The hydroxyl radical scavenging activity of RvD1 and analogues was performed in a 96-well black plate in the presence of CuCl_2_ and H_2_O_2_ in acellular conditions [44]. Briefly, 10 μM CuCl_2_ and 100 μM H_2_O_2_ were mixed in a 100 μL/well of PBS (75 mM, pH 7.4) in the presence or absence of RvD1, Analogue 1, Analogue 2, and Trolox at a concentration of 10 μM. Then, afterwards, 100 μL of DCFH-DA probe solution was added to each well in a final concentration of 20 μM. The fluorescence was measured at 0, 0.5, 1, 2, 4, and 8 h after the exposure time, using a microplate reader with fluorescence polarization (Polar Star Optima, BMG Labtech, Ghelph, Canada) set up at an excitation wavelength of 485 nm and emission wavelength of 530 nm. The results were expressed as relative fluorescence units (RFU).

### 2.4. Hyaluronic Acid Degradation Study

The hyaluronic acid degradation assay was performed as described by Chen et al. [44] with minor modifications. Briefly, a solution of HA at 1 mg/mL was treated with 10 μM CuCl_2_, 100 μM H_2_O_2_ in the presence or absence of Trolox, RvD1, or analogues at a final concentration of 10 μM. The reaction was conducted in 37 °C during 16 h. Then, 20 μg of HA was loaded into an agarose gel (2% prepared in TBE buffer) using a loading buffer containing 0.25% of bromophenol blue solubilized in formamide. After 4 h of migration at 40 volts, the HA was visualized after staining with Stain-All dissolved in 30% ethanol.

### 2.5. Hyaluronic Acid Analysis by Gel Permeation Chromatography

Gel permeation chromatography/size exclusion chromatography (GPC/SEC) measurements were performed by Two AquaGel columns (PAA-202 + PAA-206M) connected in series (PolyAnalytik, London, ON, Canada) using a Viscotek TDA305 and GPCmax. It is equipped with an oven that houses three detectors: Refractive Index (RI), Right Angle and Low Angle Light Scattering (RALS/LALS), and Four-Capillary Differential Viscometer. The measurements were carried out in 8.5 g/L NaCl in Milli-Q as the mobile phase at a flow rate of 0.7 mL/min at 30 °C. PEG 20 kDa (dn/dc = 0.132 mL/g) and dextran 72 kDa (dn/dc = 0.147 mL/g) with a concentration of 3 mg/mL were used as standards for the determination of the calibration curve. The samples were diluted using the mobile phase to a concentration of approximately 0.25 mg/mL and filtered with 0.22 µm Nylon syringe filters prior to injection. The sample concentration was approximately 2 mg/mL, and the injection load was 100 μL.

### 2.6. Computational Studies

This study was carried out using the Gaussian 09 software package. The ground-state geometry optimization of antioxidants was completed with DFT using the B3LYP/6-31 + G (d, p) to obtain the minimum on the potential energy surface. The plotting of the three-dimensional mapping of the molecular orbitals and the highest occupied molecular orbital (HOMO) and lowest unoccupied molecular orbital (LUMO) energies were calculated using the B3LYP/6-31 + G (d, p) basis set.

## 3. Results and Discussion

### 3.1. Antioxidants and Radical Scavenging Activities of Resolvin Analogues

#### 3.1.1. ORAC Assay

**Figure 4:**
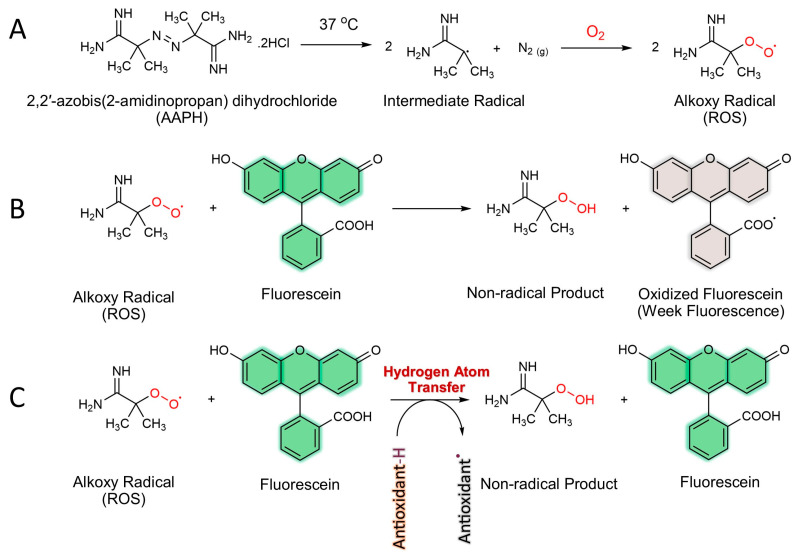
ORAC assay mechanism. (**A**) Generating ROS (alkoxy radical) by thermal decomposition of 2,2′-azobis(2-amidinopropan) dihydrochloride (AAPH) in the presence of oxygen. (**B**) Reacting ROS with fluorescein (fluorescent probe) without antioxidant compounds and production of non-fluorescent molecule. (**C**) Inhibition of ROS by antioxidants and protection of fluorescein from radical attack.

Several in vitro chemical assays have been developed and reviewed for measuring antioxidant capacity. The complicated mechanism among free radicals, substrates, and antioxidants makes it impossible to present an equation to express the kinetic order. An accurate quantification of antioxidant potential mandates the consideration of both the inhibition degree, denoting the extent of free radical scavenging, and the inhibition time, reflecting the persistence of the antioxidant effect. In this regard, the oxygen radical absorbance capacity (ORAC) assay stands out as the sole technique currently available that effectively integrates these complementary parameters into a single informative value. Thus, this method is widely used in the nutraceutical, pharmaceutical, and food industries as well as in biomedical studies [39]. In this assay, a radical-generating compound, 2,2′-azobis(2-amidinopropan) dihydrochloride (AAPH), produces biologically relevant oxygen radical species (ROS) via thermal decomposition in the presence of oxygen. As the ROS oxidizes the fluorescein, the fluorescence of the solution decreases over time. In the presence of an antioxidant, ROS can be scavenged, and the oxidation of fluorescein can be delayed until the antioxidant is depleted. Indeed, antioxidants protect fluorescein from ROS attacking by the way of a hydrogen atom transfer (HAT) process (Figure 4).

**Figure 5:**
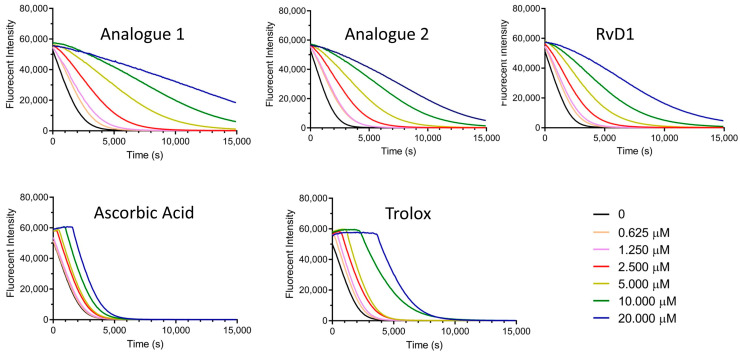
Signal curves for different antioxidant concentrations (0.625 to 20 μM) at 37 °C (normalized to initial fluorescence signals after AAPH addition).

In Figure 5, the typical time-dependent decays of fluorescence intensity of fluorescein induced by AAPH alone, or in the presence of antioxidants (final concentration range between 0.625 and 20 μM), were illustrated. After the pre-incubation of fluorescein with different concentrations of the antioxidants (including Analogue 1, Analogue 2, RvD1, ascorbic acid, and Trolox), dose-dependent inhibitions of the fluorescence decay were monitored. As seen in Figure 5, both Analogues 1 and 2 showed the ability of free radical quenching in the radical propagation cascade. According to the shape of their fluorescence decay curves, there is no clear distinction between lag time and fluorescein decay periods by increasing the concentrations of the analogues, indicating more complex scavenging kinetics. On the other hand, a very low change was observed for RvD1 by increasing the concentrations, which means low scavenging kinetics at the tested condition (Figure 5). Ascorbic acid and Trolox as controls were also tested to compare the antioxidant capacity of the samples. Although ascorbic acid is a very strong antioxidant molecule, as seen in Figure 5, it has a relatively low decay of fluorescence intensity at the tested concentration ranges. Trolox as another control showed a clear lag time, proving that it has antioxidant properties to protect fluorescein from ROS (Figure 5). As a blank control, fluorescein was exposed to excitation light at 485 nm in the absence of AAPH and antioxidants during the assay. The results proved that there is no significant fluorescence intensity change; thus, fluorescein is a stable fluorescent probe according to the analysis.

**Figure 6:**
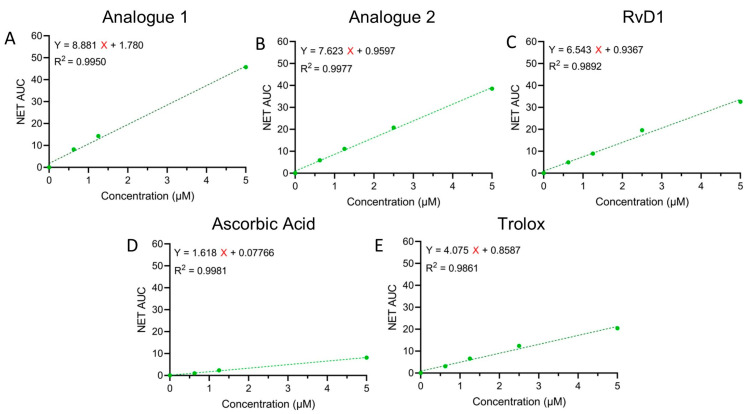
ORAC calibration curves for Analogue 1 (**A**), Analogue 2 (**B**), RvD1 (**C**), ascorbic acid (**D**), and Trolox (**E**).

The antioxidant capacity correlates to the fluorescence decay curve, which is usually represented as the area under the curve (AUC) and calculated according to Equation (1). ORAC calibration curves are depicted in Figure 6 where net AUC values were plotted against antioxidant concentrations. Trolox equivalent (TE) is a factor that presents the antioxidant capacity compared to Trolox as a standard. Based on this factor, both analogues have better antioxidant capacity compared to other tested antioxidants. Table 1 summarizes the ORAC values of all the samples expressed as μM concentration of Trolox equivalent.

Analogue 1 > Analogue 2 > RvD1 > Trolox > Ascorbic acid

#### 3.1.2. DPPH Assay

**Figure 7:**
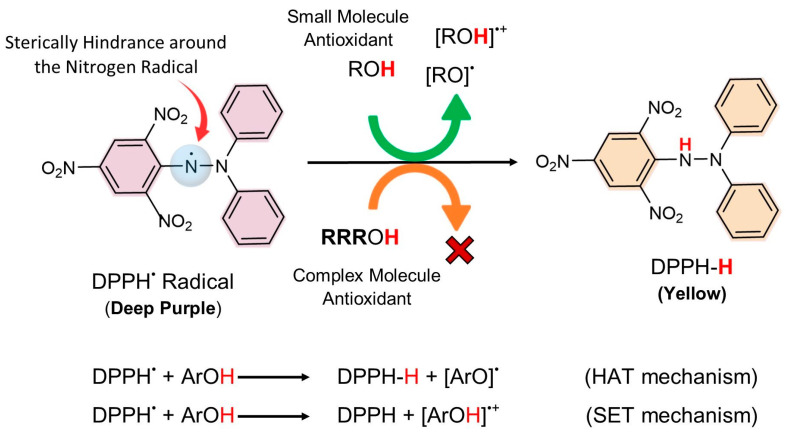
Reaction mechanism of 2,2-diphenyl-1-picrylhydrazyl (DPPH) with two different types of antioxidants (small molecules and complex molecules) via hydrogen atom transfer (HAT) or single electron transfer (SET). Low possibility of interaction between complex molecules and DPPH radical due to the steric hindrance around the radical.

DPPH assay is one of the well-known and routinely practiced assessments of the free radical scavenging potential of antioxidant molecules since it is simple, quick, and relatively inexpensive. It is considered one of the standard and easy colorimetric methods for the evaluation of the antioxidant properties of pure compounds. This assay is based on the reduction in DPPH^•^ free radicals by antioxidant species accepting either a hydrogen atom or an electron (Figure 7). The DPPH reagent is considered a stable radical and has limited similarities with peroxyl radicals [45]. Its stability originates from the delocalization of unpaired electrons on a nitrogen atom in the aromatic ring (diphenylamino group as an electron donor and picryl as an electron acceptor). In this way, the DPPH radical does not dimerize like many other free radicals. The limited space surrounding the nitrogen atom sterically hinders the addition of bulky radicals to this region. Thus, the complex molecules with hydrogen-donating sites (like RvD1 and its analogues) cannot approach the nitrogen atom and release the hydrogens there to the formation of hydrazine [46,47]. Since this assay is a colorimetric method, the reduction form of DPPH^•^ free radical (DPPH-H) can be followed by turning purple to a yellow color.

As all samples showed antioxidant activity in the ORAC assay at the range of 0.625–20 μM, the same range concentration was also chosen for the DPPH assay. Both control samples, ascorbic acid and Trolox, showed low scavenging percentages of 4.31 ± 2.74 and 3.53 ± 1.88 µM at 20 μM, respectively, whereas RvD1, Analogue 1, and Analogue 2 did not show any radical scavenging properties under the same conditions. To find the half-maximal inhibitory concentration (IC_50_) value of the samples, higher concentrations in the range of 20–80 µM were also tested. IC_50_ refers to the antioxidant concentration that scavenges 50% of the initial DPPH radicals in a specific but arbitrary time interval. The color change was monitored spectrophotometrically and utilized for the determination of parameters for antioxidant properties. In the cases of ascorbic acid and Trolox, the purple color was changed to yellow with a concomitant decrease in absorbance at 515 nm. The IC_50_ values obtained for ascorbic acid and Trolox were around 66.87 µM and 67.13 µM, respectively. On the other hand, no radical scavenging was observed for RvD1, Analogue 1, and Analogue 2 even at higher concentrations.

It has been proved that some chemicals easily react with DPPH radicals by electron transfer or by donating hydrogen atoms. In particular, phenolic compounds are the most reactive and important ones that react easily with DPPH• [48]. For instance, Trolox has a hydroxyl group on the aromatic ring, which shows good antioxidant activity via the donating hydrogen mechanism, because the formed radical on Trolox can be stabilized by the delocalization within the aromatic ring. As mentioned above, steric hindrance around the nitrogen atom of the DPPH radical inhibits all reactions, particularly the hydrogen transfer, which is necessary for the formation of a hydrogen-bonded complex. Complex molecules get in the way of each other more easily, which blocks access to DPPH radicals at low concentrations. Additionally, methanol, which is generally used as a solvent for the DPPH method, strongly binds H atoms and inhibits HAT processes. It may be concluded that the DPPH^•^ radical might react with each antioxidant via different kinetics or might not react at all due to its stability. Furthermore, the reversibility of the reaction may lead to low readings of the antioxidant capacity of many antioxidants; consequently, the antioxidant capacity of many antioxidants is likely to be wrongly estimated.

#### 3.1.3. FRAP Assay

**Figure 8:**
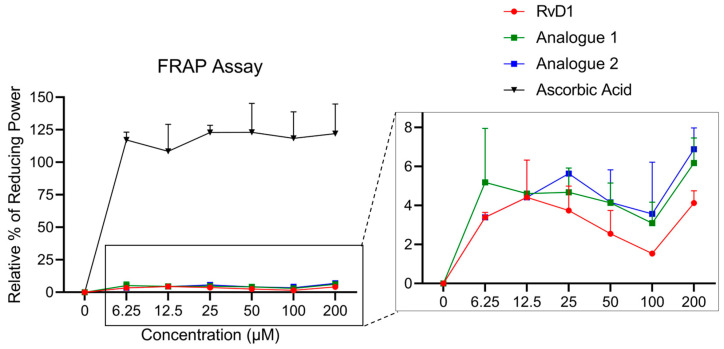
Relative percentage of reducing power of ascorbic acid, RvD1, Analogue 1, and Analogue 2 in compared to Trolox as a standard control using FRAP assay.

The FRAP assay is a rapid and automated assay that measures the electron-donating ability of an antioxidant to reduce the ferric 2,4,6-tripyridyl-s-triazine complex [Fe^3+^-(TPTZ)_2_]^3+^ to the intense, blue-colored ferrous complex [Fe^2+^-(TPTZ)_2_]^2+^ under acidic condition (pH = 3.6) [49]. Trolox equivalent (TE) is a unit of measurement used to express the antioxidant capacity of a substance relative to Trolox. All measurements for both Trolox and the sample should be made under the same conditions and concentrations [50]. The TE of ascorbic acid, RvD1, Analogue 1, and Analogue 2 were calculated based on the Trolox calibration curve. The TE values were obtained 242.53 ± 46.16, 8.65 ± 1.19, 12.71 ± 2.51, and 14.12 ± 2.14 µM at the highest treated concentration (200 µM), respectively. Additionally, the relative percentage of reducing power was also calculated at the same concentrations, and it was around 122.03% ± 22.81 for ascorbic acid at 200. However, this value for RvD1, Analogue 1, and Analogue 2 was much less, 4.12% ± 0.62, 6.17% ± 1.28, and 6.88% ± 1.09, respectively (Figure 8). All data confirmed that the RvD1 analogues showed almost two times higher antioxidant activity compared to RvD1. To figure out why the analogues with potential antioxidant abilities have very little electron-donating ability compared to controls (ascorbic acid and Trolox), the redox condition mechanism should be considered. Redox conditions refer to media dominated by oxidants (electron acceptors), the substances that can oxidize other substances (cause them to lose electrons), or reductants (electron donors), which are the substances that can reduce other substances.

There are several possibilities that the RvD1 and its analogues are not able to donate electrons easily to the ferric ion (Fe^3+^) and reduce it to a ferrous ion (Fe^2+^). The first possible reason can be related to the standard reduction potentials (E° values) of the reactants, which can vary depending on the specific reaction conditions and solvent properties. E° values represent the standard reduction potential under specific conditions of 25 °C and 1 M concentration [51]. For example, the acidic condition of the FRAP reaction (pH = 3.6) may cause the protonation of the antioxidant compounds, and as a result, it prevents the transfer of electrons from the antioxidant to the ferric ion. In the case of Trolox, the specific E° value depends on the context of the reduction reaction. At neutral pH (7.4), the relevant E° value is around −0.48 V, which indicates a stronger electron-donating tendency compared to the acidic condition (E° = −0.35 V). The E° value shifting at acidic pH is because of the protonation of the phenolic hydroxyl, affecting its electron-donating ability slightly. But in the case of ascorbic acid, the specific E° values at pH 7.4 and 4.5 are −0.28 V and −0.05 V, respectively. Although both Trolox and ascorbic acid are commonly used reference standards in the FRAP, Trolox generally has a more negative E°, indicating a stronger tendency to donate electrons and reduce ferric ions. This suggests it might show higher FRAP activity compared to ascorbic acid at neutral pH. On the other hand, from the mechanism point of view, Trolox donates a single electron via its phenolic hydroxyl group, but ascorbic acid can donate one or two electrons depending on pH and reaction conditions. Thus, the two-step electron-donating of ascorbic acid adds more complexity compared with Trolox. Regarding the RvD1 and its analogues, they showed a much lower FRAP value compared to Trolox and ascorbic acid. It can be concluded that the E° value might be less negative to be able to reduce the Fe^3+^ with an E° value of +0.77 V. To unravel the precise electron transfer processes involved in these antioxidants’ activity, a comprehensive electrochemical study is necessary.

#### 3.1.4. Hydroxyl Radical Scavenging (HRS)

**Figure 9:**
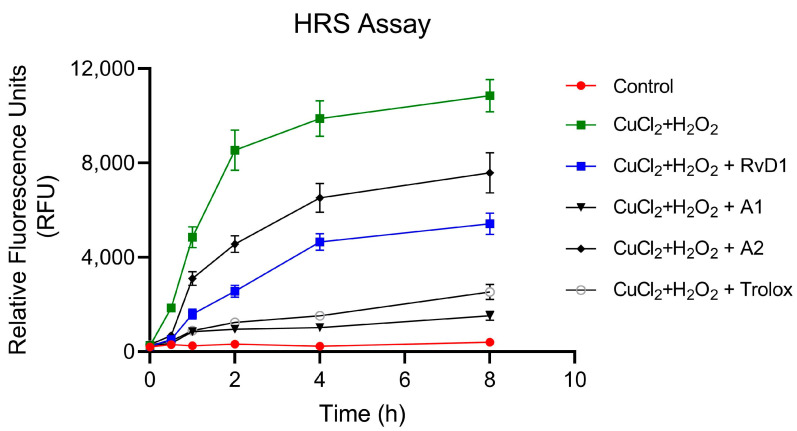
RvD1 and its analogues attenuate CuCl_2_ + H_2_O_2_-induced ROS production. ROS were induced in a black 96-well plate by the addition of CuCl_2_ (10 μM) to the H_2_O_2_ (100 μM) in the presence or absence of RvD1, Analogue 1 (A1), Analogue 2 (A2), and Trolox at 10 μM. DCFH-DA probe was added at a concentration of 10 μM. The results are expressed as relative fluorescence units (RFU) (n = 3).

This experiment was designed to investigate the antioxidant properties of RvD1 and analogues using CuCl_2_ + H_2_O_2_-induced ROS generation in the acellular environment. Trolox was used as a positive control. As illustrated in Figure 9, RvD1, Analogue 1, Analogue 2, and Trolox significantly reduced the production of ROS in a time-dependent manner. After 8 h of incubation, the generation of ROS was reduced by 30, 50, 77, and 86 by Analogue 2, RvD1, Trolox, and Analogue 1, respectively, as compared to CuCl_2_ + H_2_O_2_ alone. These findings confirm the antioxidant activity of RvD1 and our synthetic analogues.

### 3.2. Protection of Hyaluronic Acid against ROS

Hyaluronic acid (HA) is a linear polysaccharide consisting of two alternating units, β-1,4-D-glucuronic acid and β-1,3-N-acetyl-D-glucosamine. HA, found in synovial fluid, acts as an elastoviscous component that creates a friction-free surface for joint movement. The molecular weight of HA is more variable and most commonly synthesized as a high-molecular-weight polymer in normal biological fluids and tissues with an average molecular mass of approximately 1000–8000 kDa [52]. Studies have shown that the HA concentration remains relatively stable with age or tends to decline between 28 and 40 years old and remains at a low level beyond that age. Its concentration in human synovial fluid ranges from 1.5 to 3.6 mg/mL. Hence, HA with an average molecular weight of 1500 kDa was dissolved in PBS buffer solution (pH 7.4, 150 mM) with a concentration of 2 mg/mL. Then, the HA solution was incubated with CuCl_2_ + hydrogen peroxide (H_2_O_2_) without or in the presence of antioxidants (RvD1, Analogue 1, Analogue 2, and Trolox). To evaluate the radical scavenging capability of the antioxidants, the following studies were performed.

#### 3.2.1. Agarose Gel Electrophoresis

**Figure 10:**
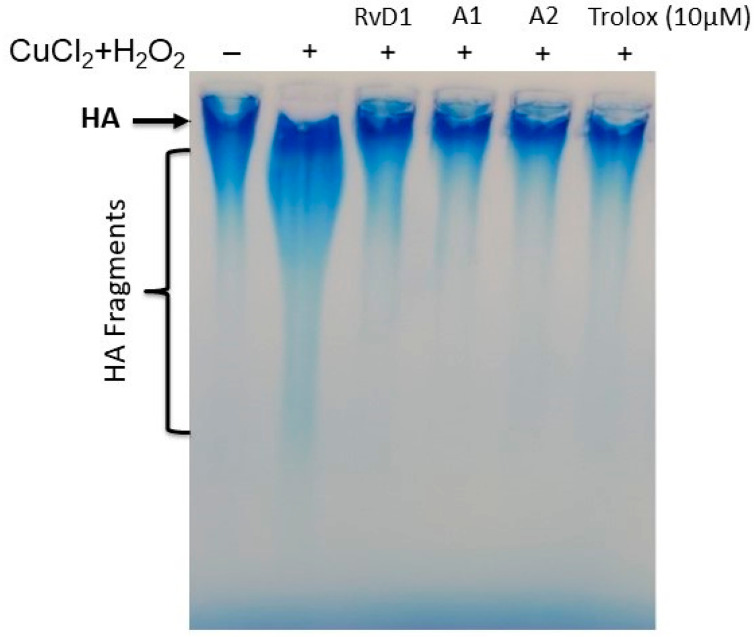
RvD1, Analogue 1 (A1), Analogue 2 (A2), and Trolox inhibit CuCl_2_ + H_2_O_2-_induced hyaluronic acid (HA) degradation. A solution of commercial HA (1 mg/mL, MW: 1000–2000 kDa) was exposed to 10 μM CuCl_2_ and 100 μM H_2_O_2_ for 16 h in the presence or absence of RvD1, analogues or Trolox at 10 μM. Samples were then loaded in agarose gel/1X TBE, and then hyaluronic acid was revealed using a stain-all solution (n = 3).

Here, the capacity of RvD1 and analogues was investigated to block ROS-induced HA degradation. To do so, the polymer was treated with RvD1 and analogues and then after with CuCl_2_ + H_2_O_2_ for 16 h. The degradation of HA was evaluated by gel agarose electrophoresis. As indicated in Figure 10, the addition of RvD1 and analogues protected CuCl_2_ + H_2_O_2-_induced HA fragmentation and preserved its integrity of HA as compared to the untreated HA.

#### 3.2.2. Gel Permeation Chromatography

**Figure 11:**
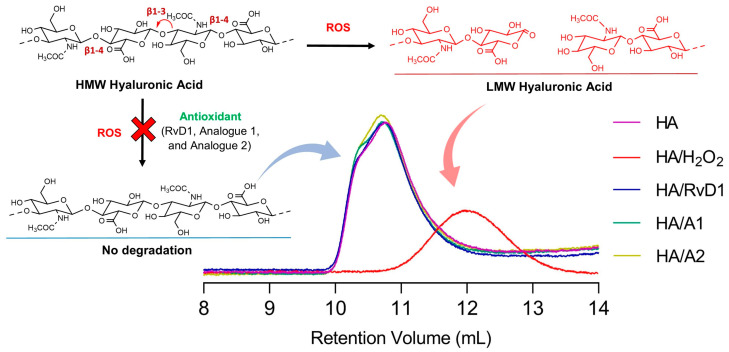
GPC/SEC chromatogram. Molecular weight comparison of HA after incubation with CuCl_2_ + H_2_O_2_ without or in the presence of RvD1 and RvD1 analogues as radical scavengers.

Based on GPC results (Figure 11), the average molecular weight of HA was about 1,160,000 Da with a dispersity (M_w_/M_n_) of 1.103 and intrinsic viscosity of 13.5227. These values were changed significantly after the incubation of HA with hydrogen peroxide. The GPC chromatogram showed a symmetric broad peak with an average molecular weight of 217,211 Da, a dispersity value of 1.651, and an intrinsic viscosity of 4.7605. The degradation is because the hydroxyl radical can abstract hydrogen atoms at all ring C-H bonds except C-2 of N-acetyl hexosamine. The abstraction of hydrogen atoms generates carbon-center radicals which undergo a β-scission reaction, resulting in the breakdown of the HA chains. ROS-mediated HA degradation occurs randomly and generates not only glycosidic linkage cleavage but also the possibility of other structural changes, including ring opening and the occurrence of different conformational characteristics. However, the RvD1 analogues as antioxidants scavenged the formed free radical and protected the HA from degradation. The GPC results showed that the average molecular weights of HA were almost the same as the control (free HA), which obtained 1,031,000, 1,043,000, and 1,026,000 Da for RvD1, Analogue 1, and Analogue 2, respectively. It can be concluded that the analogues have very good potential to protect HA from ROS degradation. All data obtained are summarized in Table 2 below, which includes individual injection results.

### 3.3. Computational Study

Experimental methods have been used to determine the total antioxidant activity of samples. However, for a comprehensive evaluation of the radical scavenging activity of samples, computational methods are required. In this study, two concepts were used to analyze the antioxidant activity of samples: hydrogen atom transfer (HAT, which is based on the electronic and thermal enthalpy) and single-electron transfer (which is based on the frontier orbital energies of neutral molecules). To attain this, some parameters were calculated, such as the energy gap (∆E), ionization potential (IP), bond dissociation enthalpy (BDE), hardness, softness, and electronegativity.

#### 3.3.1. Geometry Optimization of Samples

**Figure 12:**
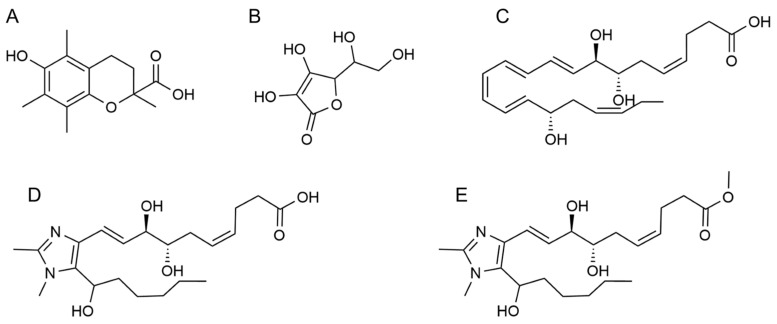
Molecular structure of Trolox (**A**), ascorbic acid (**B**), RvD1 (**C**), Analogue 1 (**D**) and Analogue 2 (**E**).

**Figure 13:**
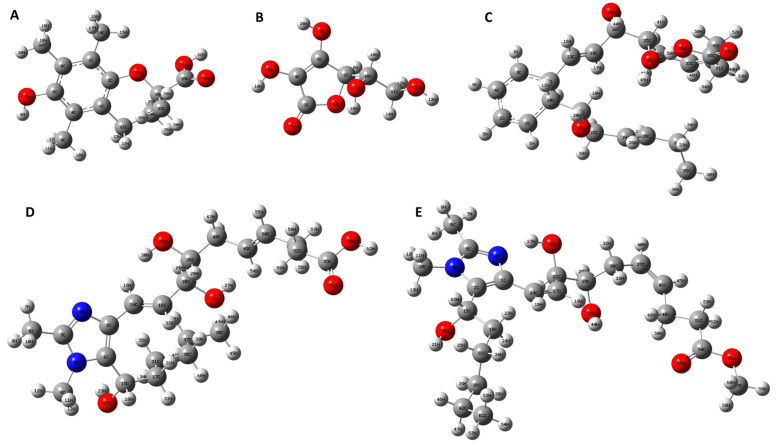
Geometrical molecular structure of Trolox (**A**), ascorbic acid (**B**), RvD1 (**C**), Analogue 1 (**D**), and Analogue 2 (**E**) using Gaussian 09—ground states optimized at the B3LY/6-31G (d, p) function (all structures were also provided separately in the supporting information).

Geometry optimization is one of the most essential tasks in theoretical chemistry. It is a method to predict the three-dimensional arrangement of the atoms in a molecule employing the minimization of model energy. The molecular structures of all tested antioxidant samples are depicted in Figure 12 (designed by ChemDraw software version 12.0.0). As shown, the molecules have several functional groups such as aromatic ring, hydroxyl (O-H), ester (O=C-OR), carboxylic acid (O=C-OH), and carbon–carbon-conjugated double (C=C) bonds. Among all functional groups, there are only four main structural motifs that contribute to the antioxidant activity of small molecules. These functional motifs include hydroxyl groups (-OH), amino groups (-NH), thiol groups (-SH), and isoprenoid groups. In this study, all the tested antioxidants have hydroxyl functional groups in common; thus, comparing the bond strength of their O-H can be a way to estimate their antioxidant potential. It was found that the weaker O-H bond shows a higher antioxidant potential. Therefore, to obtain more data about geometrical molecular structure and their bonds, the DFT method with B3LY/6-31G (d, p) function was performed for optimization and analysis of the antioxidant samples, including Trolox, ascorbic acid, RvD1, Analogue 1, and Analogue 2 (Figure 13). The bond lengths obtained through the optimized molecular structure of different samples are useful data to explain the HAT.

Within the Trolox structure, there are two hydroxyls (O-H) bonds, and the bond lengths related to phenol (O7-H8) and carboxylic acid (O31-H32) are 0.9741 and 0.9821 Å, respectively. Based on the shorter the bond, the stronger the bond, the hydroxyl bond in carboxylic acid should be weaker because of the higher bond length. Since the bond length difference is very low (around > 1%), the determination of the better hydrogen donating cannot be very precise. In the case of ascorbic acid, its structure includes four hydroxyl groups, and the shortest O-H bonds are O12-H13 and O8-H20 with a length equal to 0.9772 Å and 0.9791 Å. In contrast, the longest value belongs to O9-H19 and O17-H18 with a length equal to 0.9797 Å. As a result, O9 and O17 in ascorbic acid and O7 in Trolox can be supposed as the active sites for hydrogen donating. As seen in Table 3, the longer O-H bond lengths in RvD1, Analogue 1, and Analogue 2 were related to O56-H58, O60-H62, and O26-H27 with the bond lengths of 0.9996, 0.9821, and 0.9981 Å, respectively. Interestingly, the O-H bond related to the carboxylic bond showed a higher bond length compared to another hydroxyl bond in RvD1 and Analogue 1. As the number and type of hydroxyl group in the tested molecules are different, comparing the hydrogen-donating properties of the molecules is not possible. Therefore, the average bond length of the hydroxyl group was calculated to show the order of the antioxidant capacity for Trolox, ascorbic acid, RvD1, Analogue 1, and Analogue 2 (with average bond lengths of 0.9781, 0.9789, 0.9856, 0.9801, and 0.9856 Å, respectively).

Analogue 2 = RvD1 > Analogue 1 > Ascorbic acid > Trolox

#### 3.3.2. Bond Dissociation Enthalpy

Hydrogen Atom Transfer (HAT) is a crucial mechanism related to the antiradical activity of antioxidants. It is based on transferring hydrogen atoms with a homolytic dissociation of the O-H bond to free radicals. The antioxidant capacity of the HAT mechanism can be described by the bond dissociation enthalpy (BDE) parameter.

BDE = H (ArO^•^) + H (H^•^) − H (ArOH) (6)

where H (ArOH) is the enthalpy of the neutral molecule, H (ArO^•^) is the enthalpy of the radical generated via H atom abstraction, and H (H^•^) is the enthalpy of the H atom. The BDE values represent the energy necessary for cleaving the O-H bonds. The BDE values of all O-H bonds of samples are listed in Table 4. The antioxidant with the lowest BDE shows better antiradical properties. Table 4 shows clearly that the BDE value of O7-H8 of Trolox is the lowest in comparison with other hydroxyl groups in the gas phase. This means that the abstraction of the H atom from O7-H8 of Trolox is easier than from other H atoms, which indicates that the radical created from this abstraction is more stable than other radicals. Moreover, among RvD1 and its analogues, the BDE value of the O22-H23 bond in the structure of Analogue 1 is the lowest, which indicates its higher antioxidant activity. According to the BDE values, the antioxidant activity of antioxidant samples decreases in the following order:

Trolox > Ascorbic acid > Analogue 1 > Analogue 2 > RvD1

All calculations of the neutral compounds and their radicals were calculated using Gaussian 09 software.

#### 3.3.3. Ionization Potential

Single electron transfer (SET) is another main mechanism of antioxidants, in which a single electron transfers from the antioxidant to the free radical. The antioxidant capacity of the SET mechanism can be described by the ionization potential (IP) parameter.

IP = H (ArOH^•+^) + H (e^−^) − H (ArOH) (7)

where H (ArOH) is the enthalpy of the neutral molecule, H (ArOH^•+^) is the enthalpy of the radical cation, and H (e^−^) is the enthalpy of a single electron. The ionization potential (IP) shows the ease of electron removal from antioxidant samples. An antioxidant sample with a higher IP value shows a lower electron donation tendency; therefore, an electron is hardly removed from the HOMO orbital of the neutral form of the compound. On the other hand, a compound with a lower IP value has a higher electron transfer capacity. The IP values of samples are listed in Table 5. According to the data in Table 5, Trolox has the lowest ionization potential, so an electron can be easily removed from the HOMO orbital of neutral Trolox. In contrast, ascorbic acid has the highest IP, which indicates its low antioxidant activity via the SET mechanism in comparison to other samples. Concerning RvD1 and its analogues, Analogue 2 represents better electron-donating properties. According to the IP values, the antioxidant activity of antioxidant samples decreases in the following order:

Trolox > Analogue 2 > Analogue 1 > RvD1 > Ascorbic acid

#### 3.3.4. Frontier Molecular Orbitals

The electronic properties of structures such as HOMO and LUMO energies and energy gaps (∆E) of compounds can be evaluated using frontier molecular orbital theory. The HOMO (highest occupied molecular orbital) and LUMO (lowest unoccupied molecular orbital) are significant parameters that correlate with the antiradical activity of the samples. The HOMO has electron-donating ability; therefore, it is easy to remove electrons from this orbital. However, the LUMO has electron-accepting ability, and it is easy for this orbital to accept electrons. To be more precise, in a compound with a higher HOMO value, the donation of electrons is easier, and as a result, it may show better antioxidant activity. The electronic density distribution in the orbital permits prediction of the most probable sites in a molecule that can be easily attacked by free radicals and other reactive agents.

**Figure 14:**
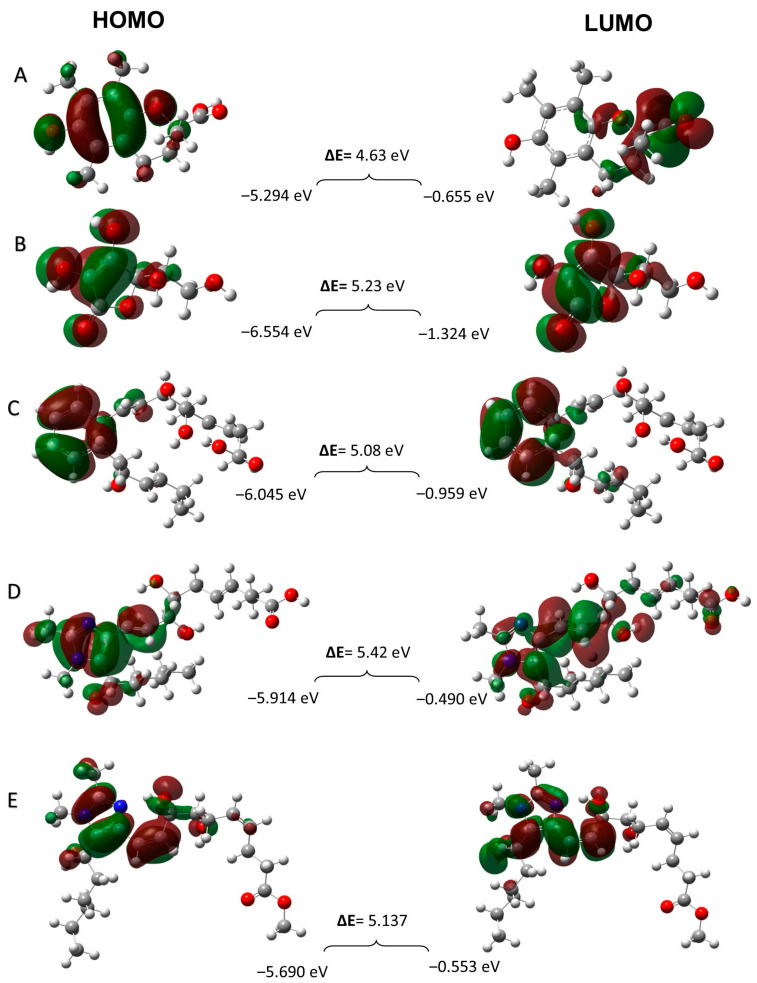
The energies and distributions of the HOMO and LUMO orbitals: Trolox (**A**), ascorbic acid (**B**), RvD1 (**C**), Analogue 1 (**D**), and Analogue 2 (**E**).

The HOMO and LUMO energies and the ∆E (LUMO-HOMO) of all antioxidant samples with the B3LYP/6-31 + G (d, p) basis set are shown in Figure 14. The energy of HOMO is related to the electron-donating ability of an antioxidant; however, the shape of HOMO shows the sites for free radical attack. As seen in Figure 14A, the HOMO of Trolox is localized over the whole molecule except for the carboxylic acid functional group. The results of HOMO and LUMO energies for ascorbic acid (Figure 14B) showed that the HOMO is localized on O8-H20, O9-H19, and the lactone ring, while the LUMO is not localized on the lactone ring but is very close to it. In the case of RvD1 (Figure 14C) and its two analogues (Figure 14D,E), the HOMOs are localized on a conjugated double bond and imidazole ring, respectively. Theoretically, the energies of HOMO orbitals are a great indicator of the scavenging activity of samples, relating to their electron-donating ability. Thus, it can be concluded that these regions are the most probable sites for free radical attack. Based on all the above-mentioned results, it can be perceived that the HOMO energy of Trolox (−5.294 eV) is the highest, and the lowest HOMO is related to ascorbic acid (−6.554 eV), which means that the radical-scavenging ability of Trolox should be much better than ascorbic acid as controls. Additionally, the HOMO energy values for the RvD1, Analogue 1, and Analogue 2 are −6.045, −5.914, and −5.690 eV, respectively. Based on these results, Analogue 2 with the highest HOMO energy is more probable to donate electrons than RvD1 and Analogue 1. According to the HOMO energy values in all samples, it can be assumed that the radical-scavenging ability based on HOMO energy arrangement is as follows. This evidence confirms the IP results, which are shown above.

Trolox > Analogue 2 > Analogue 1 > RvD1 > Ascorbic acid

#### 3.3.5. Quantum Chemical Parameters

The chemical activity of compounds can be calculated by the frontier molecular orbitals energies (HOMO and LUMO). Hardness (η), softness (σ), electronegativity (χ), chemical potential (µ), and electrophilicity (ω) are the quantum chemical parameters to evaluate the chemical behavior of compounds. The polarizability of molecules is an important factor which is associated with the band gap energy. A molecule with high band gap energy shows less polarizability. Generally, soft molecules are more strongly polarizable than hard molecules, so the softness value shows the ability to transfer electrons. On the other hand, the hardness value is related to the resistance of the molecule to lose electrons. Therefore, the hard molecules have a high HOMO–LUMO gap (∆E _gap_) which tends to have high stability and low chemical reactivity with radicals. Overall, it may be said that the soft molecules have a low ∆E _gap_, low stability, and high reactivity leading to higher antioxidant activity [53].

Electronegativity (χ) is the tendency of a molecule to attract electrons, which is a factor to compare the antioxidant activity. According to the results, lower electronegativity is related to Trolox, which means that it has more ability to donate electrons rather than obtain them. Electrophilicity (ω) is the ability of a molecule to take up electrons from the surroundings and depicts the electron-accepting ability of species. Compounds with lower electrophilicity indexes are more likely to donate electrons and exhibit higher antioxidant activity. The lowest electrophilicity is related to Analogue 1 (the strongest antioxidant among under-studied samples), and the highest electrophilicity is related to ascorbic acid (the weakest antioxidant among under-studied samples). The formula of all parameters is based on the Koopmans theorem’s and is shown below. Table 6 and Table 7 show the equation for quantum chemical parameters for the studied molecules and the calculated value for quantum chemical parameters, respectively.

#### 3.3.6. Molecular Electrostatic Potential (MEP)

**Figure 15:**
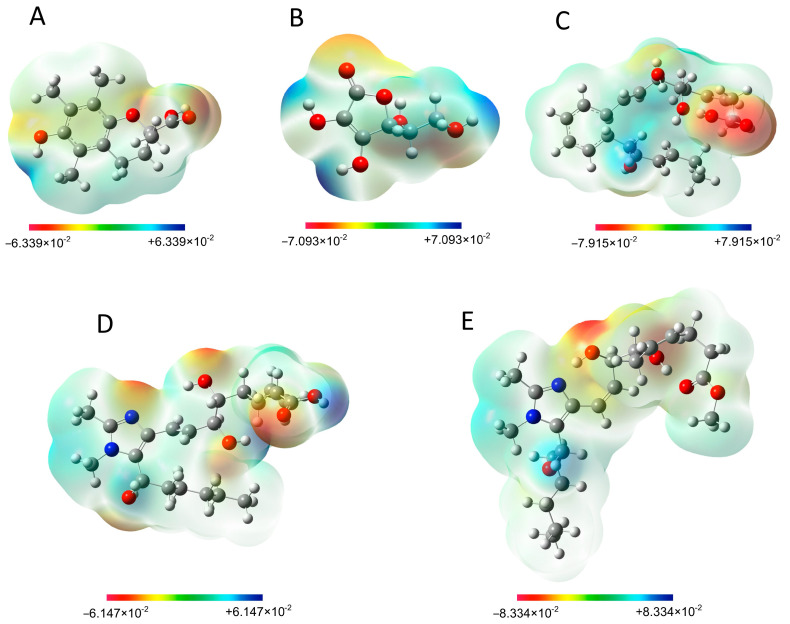
The molecular electrostatic potential surface (ESP) of Trolox (**A**), ascorbic acid (**B**), RvD1 (**C**), Analogue 1 (**D**), and Analogue 2 (**E**).

The electrostatic potential map provides useful information related to the charge distribution of a molecule and information about the active sites of a molecule toward nucleophilic and electrophilic attack [54]. The MEP maps of antioxidant samples are shown in Figure 15. The electrostatic potential values of different sites are specified by different colors. The area with red color represents the most negative electrostatic potential (ESP, signifying an electron-rich region for electrophilic attack), the blue color displays the most positive electrostatic potential (signifying an electron-deficient region for nucleophilic attack, such as radicals and charged molecules), and the green color illustrates the neutral electrostatic potential. Therefore, the negative ESP spots are significantly concentrated over oxygen atoms in all samples and nitrogen atoms in Analogues 1 and 2 (abundance of electrons in this region, active sites for electrophilic attack), and the positive ESP spots are mainly concentrated over some hydrogen atoms (absence of electrons in this region, active sites for nucleophilic attack) [55]. The most electronegative area (red region) is responsible for donating an electron, and the most electropositive area (blue region) is responsible for donating a proton.

#### 3.3.7. Mulliken Charge Distribution

The Mulliken charge distribution of all samples is calculated on B3LYP at 6-31 G (d, p) level theory. Mulliken charge values for the atoms of all antioxidant samples are shown in Figures S9–S13. In all samples, hydrogen atoms are positively charged; however, nitrogen and oxygen atoms acquire a negative Mulliken atomic charge due to their electronegativity. Regarding carbon atoms, they are influenced by their substituents. Hence, all carbon atoms are negatively charged except for those which are boned with nitrogen and oxygen atoms. This is because electronegative atoms (N and O) pull out the partial charge from carbon atoms and make carbon atoms acquire a positive atomic charge.

## 4. Conclusions

Oxidative stress is closely linked to osteoarthritis progression and is a crucial target for therapy. Previous studies have reported that antioxidant treatment could reduce the degradation of the cartilage matrix and delay osteoarthritis progression. In the present study, new imidazole-derived RvD1 analogues as antioxidants with good potential for OA treatment were compared with commercially available RvD1. The in vitro chemical assay including ORAC, HRS, and FRAP assays showed that imidazole-derived RvD1 analogues have higher antioxidant capacities than commercially available RvD1. Interestingly, based on the ORAC assay, the analogues demonstrated four and there times more antioxidant capacity than ascorbic acid and Trolox, respectively. Agarose gel electrophoresis results showed that the analogues have a good ability to protect HMW HA from degradation by ROS. The GPC analysis also confirmed the obtained results. To find the hydrogen and electron-donating sites of the RvD1 analogues, a computational study was employed. This study utilized Gaussian 09 for density functional theory (DFT) calculations with the B3LYP/6-31 G (d, p) basis set. This approach allowed for the calculation of key parameters: bond dissociation enthalpy (BDE), ionization potential (IP), and highest occupied molecular orbital (HOMO) energy, along with other quantum chemical parameters. The bond dissociation energy results confirmed that RvD1 has the strongest bond, making its analogues better hydrogen donors (Analogue 1 > Analogue 2 > RvD1). Furthermore, the ionization potential trend and HOMO energy of the RvD1 analogues reflect a greater ease of electron donation, leading to superior antioxidant properties of the analogues compared to RvD1 (Analogue 2 > Analogue 1 > RvD1). All calculated values support the enhanced electron-donating character of the analogues. These computational findings are in excellent agreement with the experimental results from ORAC, FRAP, and HRS assays. Overall, the combined evidence strongly supports the potential of our newly designed imidazole-derived RvD1 analogues as effective antioxidant agents for OA treatment. This encourages us to utilize them for further in vivo studies and develop antioxidant delivery systems for the treatment of inflammatory diseases.

## Figures and Tables

**Table 1:** The Trolox equivalent (TE) values for antioxidant samples.

Antioxidant	Analogue 1	Analogue 2	RvD1	Ascorbic Acid	Trolox
TE Value	2.17	1.87	1.60	0.40	1

**Table 2:** GPC/SEC data of hyaluronic acid degradation with H_2_O_2_ without or in the presence of RvD1 and its analogues (triple detection GPC results of the samples were calculated using a Refractive Index Increment (dn/dc) value of 0.158 mL/g for hyaluronic acid in water).

Sample	V_p_ ^1^ (mL)	M_w_ (Da)	M_n_ (Da)	M_w_/M_n_ ^2^ (-)	IV ^3^ (mg/mL)
Hyaluronic Acid	10.76	1,160,000	1,052,000	1.103	13.5227
Hyaluronic Acid/H_2_O_2_	11.97	217,211	131,579	1.651	4.7605
Hyaluronic Acid/H_2_O_2_ + RvD1	10.75	1,113,000	1,031,000	1.079	13.9877
Hyaluronic Acid/H_2_O_2_ + A1	10.71	1,126,000	1,043,000	1.079	13.9109
Hyaluronic Acid/H_2_O_2_ + A2	10.70	1,112,000	1,026,000	1.084	13.6087

^1^ Peak Retention Volume. ^2^ Polydispersity Index. ^3^ Intrinsic viscosity.

**Table 3:** Structural parameters of Trolox, ascorbic acid, RvD1, Analogue 1, and Analogue 2: the bond length of the hydroxyl group (O-H).

Antioxidants	Bond	Bond Length (Å) *
Trolox	O7-H8	0.9741
	O31-H32	0.9821
Ascorbic acid	O8-H20	0.9791
	O9-H19	0.9797
	O12-H13	0.9772
	O17-H18	0.9797
RvD1	O19-H20	0.9786
	O42-H44	0.9821
	O43-H45	0.9824
	O56-H58	0.9996
Analogue 1	O22-H23	0.9795
	O25-H27	0.9780
	O29-H30	0.9808
	O60-H62	0.9821
Analogue 2	O20-H21	0.9795
	O26-H27	0.9981
	O43-H44	0.9793

* (1 Å = 10^−10^ m).

**Table 4:** O-H bond dissociation enthalpy (BDE) of samples (kJ/mol).

Antioxidants	Bond	BDE
Trolox	O7-H8	280.9
	O31-H32	346.9
Ascorbic acid	O8-H20	290.3
	O9-H19	293.9
	O12-H13	383.6
	O17-H18	380.5
RvD1	O19-H20	471.4
	O42-H44	421.6
	O43-H45	385.4
	O56-H58	415.0
Analogue 1	O22-H23	375.4
	O25-H27	380.5
	O29-H30	401.9
	O60-H62	411.2
Analogue 2	O20-H21	386.2
	O26-H27	403.0
	O43-H44	378.5

**Table 5:** Ionization potential (IP) of samples (kJ/mol).

Antioxidants	IP
Trolox	778.9
Ascorbic Acid	932.2
RvD1	875.3
Analogue 1	825.6
Analogue 2	806.1

**Table 6:** The equations for calculation of quantum chemical parameters of antioxidants.

Hardness (eV)	Softness (eV)	Electronegativity (eV)	Electrophilicity (eV)	Chemical Potential (eV)
$\eta =\frac{EL-EH}{2}$	$\sigma =\frac{1}{\eta }$	$\chi =\frac{-(EH+EL)}{2}$	$\omega =\frac{\chi 2}{2\eta }$	µ=−χ

**Table 7:** The calculation of quantum chemical parameters for antioxidants: hardness (η), softness (σ), electronegativity (χ), electrophilicity (ω), and chemical potential (µ).

Antioxidants	E_HOMO_	E_LUMO_	η	σ	χ	ω	µ
Trolox	−5.294	−0.655	2.31	0.432	2.974	1.914	−2.974
Ascorbic acid	−6.554	−1.324	2.61	0.383	3.938	2.970	−3.938
RvD1	−6.045	−0.959	2.54	0.393	3.500	2.411	−3.500
Analogue 1	−5.914	−0.490	2.71	0.369	3.201	1.890	−3.201
Analogue 2	−5.690	−0.553	2.56	0.390	3.121	1.902	−3.121

## Data Availability

Data are contained within the article and Supplementary Materials.

## Supplementary Materials

The following supporting information can be downloaded at https://www.mdpi.com/article/10.3390/antiox13040386/s1, Figures S1–S4: ^1^H NMR spectrum, ^13^C NMR spectrum, LCMS chromatogram, and Mass spectrum Analogue 1, respectively; Figures S5–S8: ^1^H NMR spectrum, ^13^C NMR spectrum, LCMS chromatogram, and Mass spectrum Analogue 2, respectively; Figures S9–S13: Mulliken charge distribution for Trolox, Ascorbic Acid, RvD1, Analogue 1, and Analogue 2, respectively.
