# Supplementary material for: Development of New Resolvin D1 Analogues for Osteoarthritis Therapy: Acellular and Computational Approaches to Study Their Antioxidant Activities
Source: Antioxidants (Basel). 2024 Mar 22;13(4):386. doi: 10.3390/antiox13040386 (PMC11047542; doi:10.3390/antiox13040386)
Supplement: Supplementary file 1 [file antioxidants-13-00386-s001.zip › antioxidants-2903947-supplementary.pdf]

Supplementary information

Development of New Resolvin D1 Analogues: Acellular and Computational Approaches to Study Their Antioxidant Activities

Zahra Kariminezhad[1], Mahdi Rahimi[1], Julio Fernandes[1], René Maltais[2], Jean-Yves Sancéau[2], Donald Poirier[2,3], Hassan Fahmi[4], Mohamed Benderdour[1*]

[1] Orthopedic Research Laboratory, Hôpital du Sacré-Cœur de Montréal, Université de Montréal, Montréal, QC, Canada.
[2] Department of Molecular Medicine, Faculty of Medicine, Université Laval, Québec, QC, Canada.
[3] Organic Synthesis Service, Medicinal Chemistry Platform, CHU de Québec Research Center-Université Laval, Québec, QC, Canada.
[4] Osteoarthritis Research Unit, University of Montreal Hospital Research Center (CRCHUM), Montreal, QC, Canada
* Correspondance email: mohamed.benderdour@umontreal.ca; Tel.: (514 338- 2222 #3279)

RvD1 analogue 1:

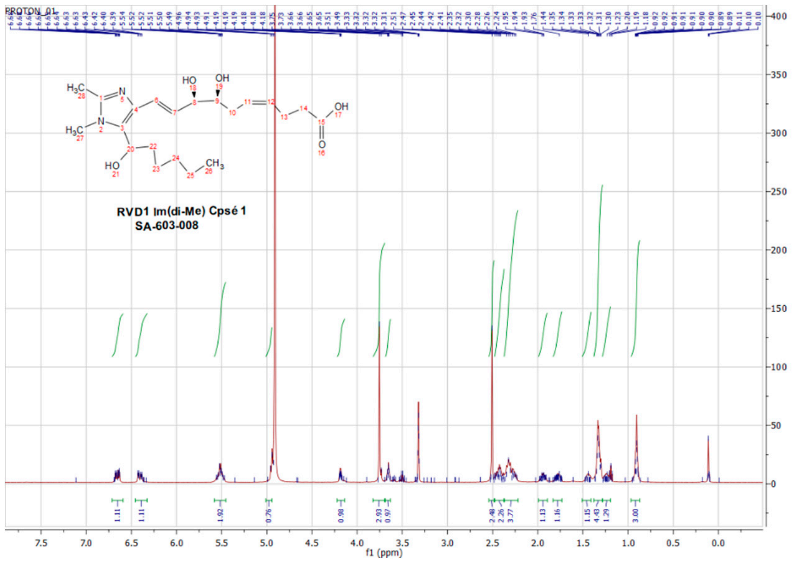

Figure S1: ^{1}H NMR spectrum analogue 1

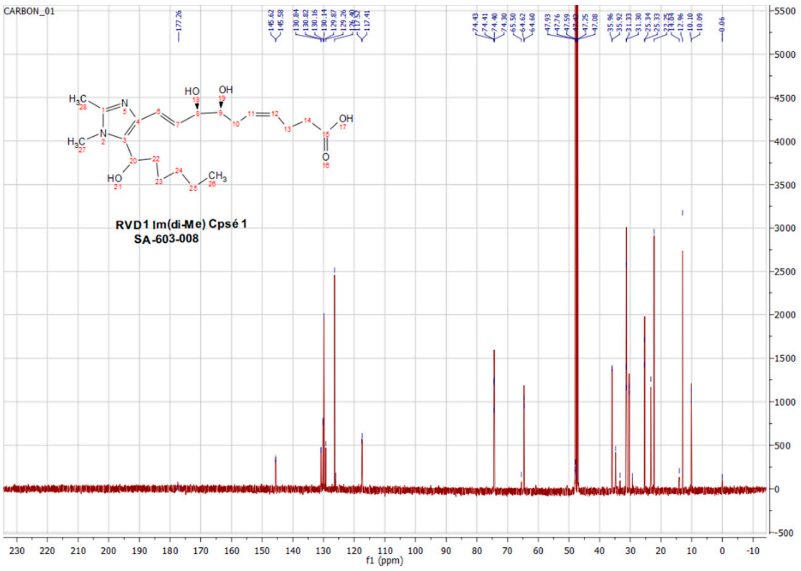

Figure S2: ^{13}C NMR spectrum analogue 1

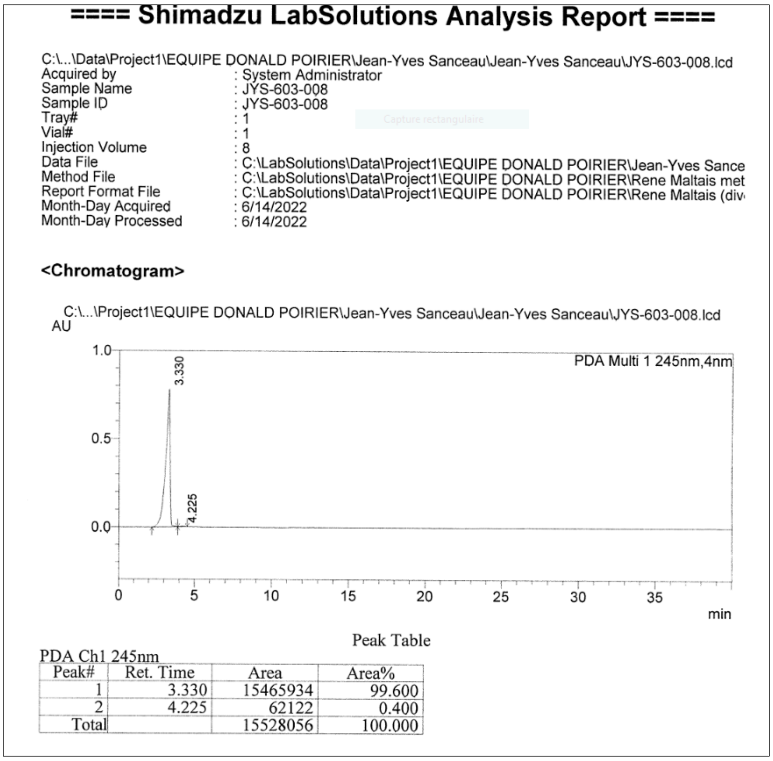

==== Shimadzu LabSolutions Analysis Report ====

C:\...\Data\Project1\EQUIPE DONALD POIRIER\Jean-Yves Sanceau\Jean-Yves Sanceau\JYS-603-008.lcd

Acquired by	: System Administrator
Sample Name	: JYS-603-008
Sample ID	: JYS-603-008
Tray#	: 1
Vial#	: 1
Injection Volume	: 8
Data File	: C:\LabSolutions\Data\Project1\EQUIPE DONALD POIRIER\Jean-Yves Sance
Method File	: C:\LabSolutions\Data\Project1\EQUIPE DONALD POIRIER\Rene Maltais met
Report Format File	: C:\LabSolutions\Data\Project1\EQUIPE DONALD POIRIER\Rene Maltais (div
Month-Day Acquired	: 6/14/2022
Month-Day Processed	: 6/14/2022

<Chromatogram>

Peak Table

PDA Ch1 245nm

Peak#	Ret. Time	Area	Area%
1	3.330	15465934	99.600
2	4.225	62122	0.400
Total		15528056	100.000

Figure S3: LCMS chromatogram analogue 1

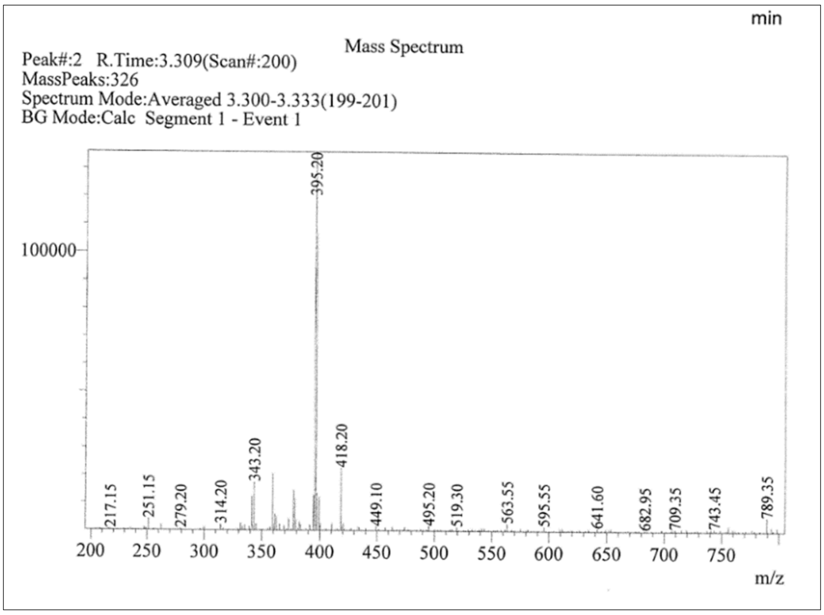

Figure S4: Mass spectrum analogue 1

RvD1 analogue 2:

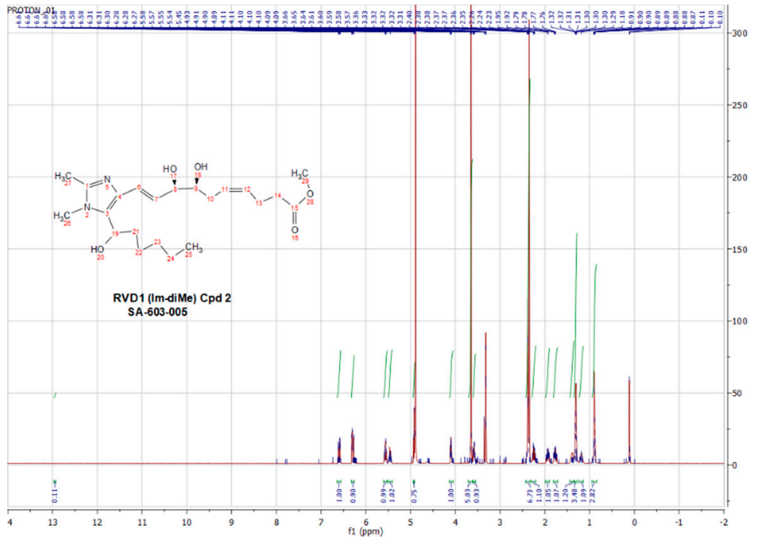

Figure S5: ^{1}H NMR spectrum analogue 2

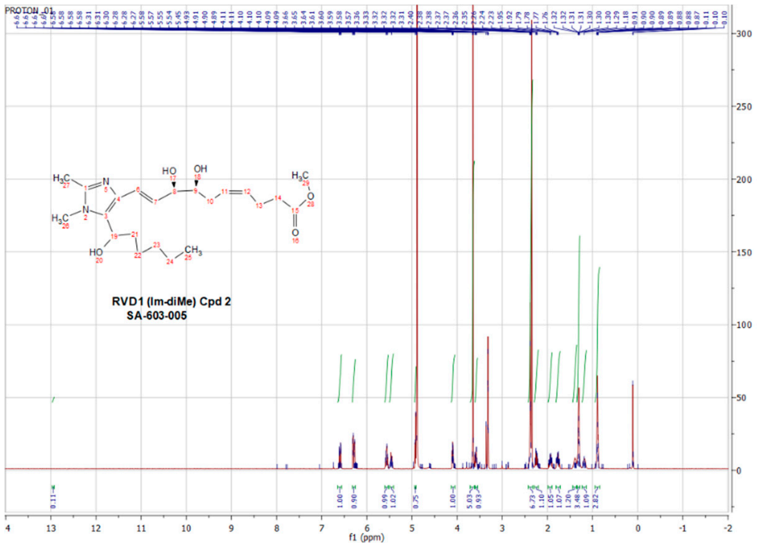

Figure S6: ^{13}C NMR spectrum analogue 2

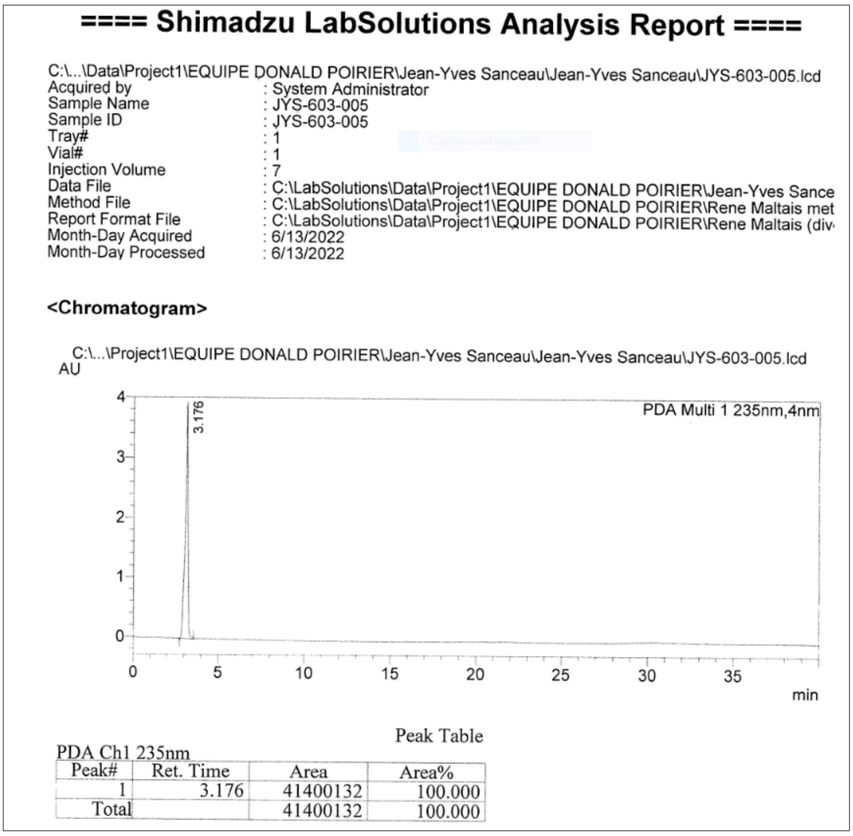

==== Shimadzu LabSolutions Analysis Report ====

C:\...\Data\Project1\EQUIPE DONALD POIRIER\Jean-Yves Sanceau\Jean-Yves Sanceau\JYS-603-005.lcd

Acquired by	: System Administrator
Sample Name	: JYS-603-005
Sample ID	: JYS-603-005
Tray#	: 1
Vial#	: 1
Injection Volume	: 7
Data File	: C:\LabSolutions\Data\Project1\EQUIPE DONALD POIRIER\Jean-Yves Sance
Method File	: C:\LabSolutions\Data\Project1\EQUIPE DONALD POIRIER\Rene Maltais met
Report Format File	: C:\LabSolutions\Data\Project1\EQUIPE DONALD POIRIER\Rene Maltais (div
Month-Day Acquired	: 6/13/2022
Month-Day Processed	: 6/13/2022

<Chromatogram>

Peak Table

PDA Ch1 235nm

Peak#	Ret. Time	Area	Area%
1	3.176	41400132	100.000
Total		41400132	100.000

Figure S7: LCMS chromatogram analogue 2

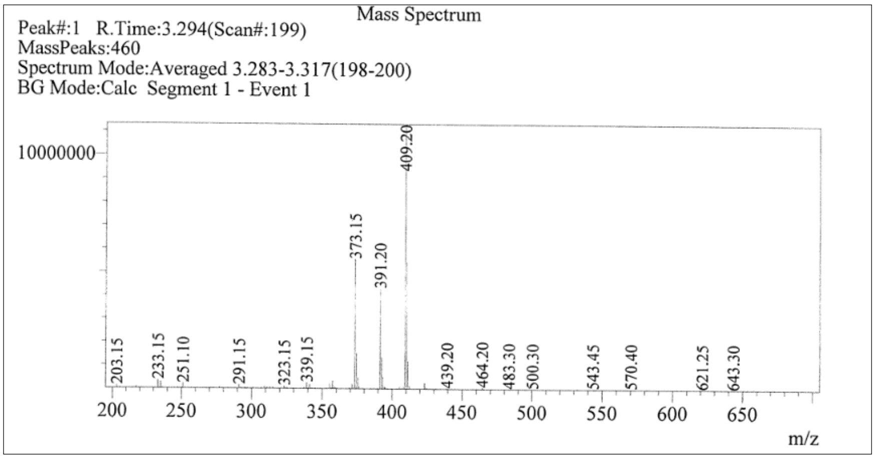

Figure S8: Mass spectrum analogue 2

Mulliken Charge Distribution:

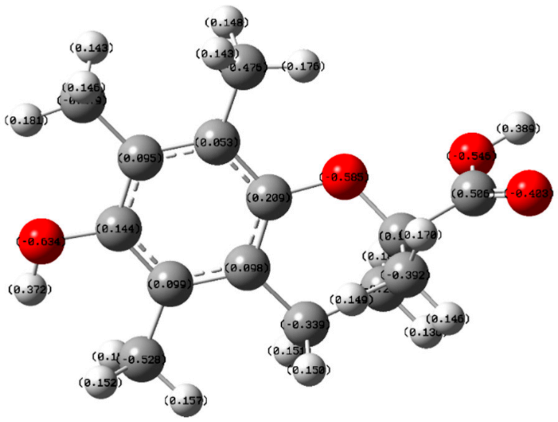

Figure S9: Mulliken charge distribution for Trolox

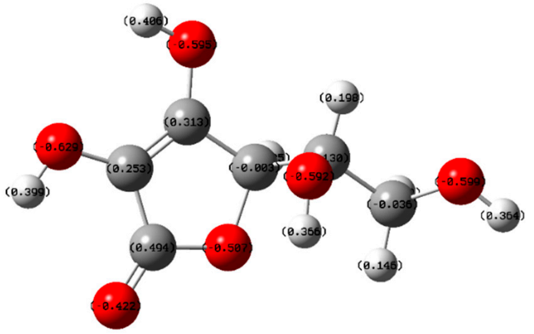

Figure S10: Mulliken charge distribution for Ascorbic Acid

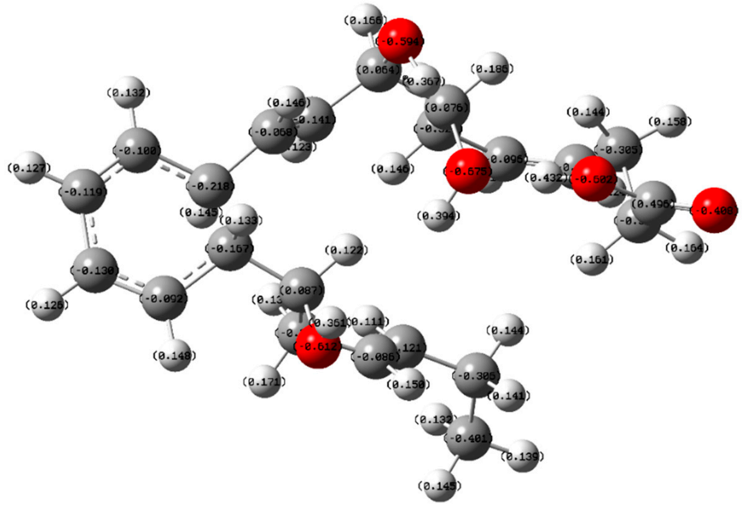

Figure S11: Mulliken charge distribution for ResolvinD1

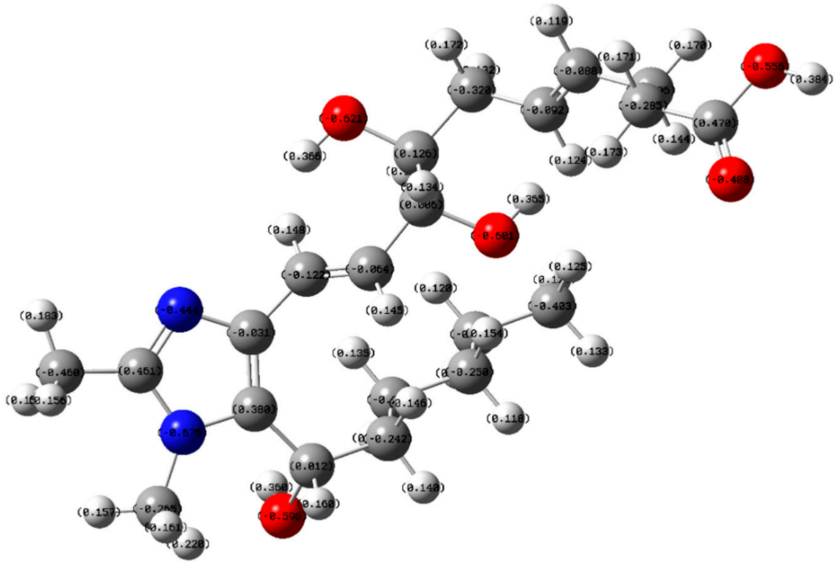

Figure S12: Mulliken charge distribution for analogue 1

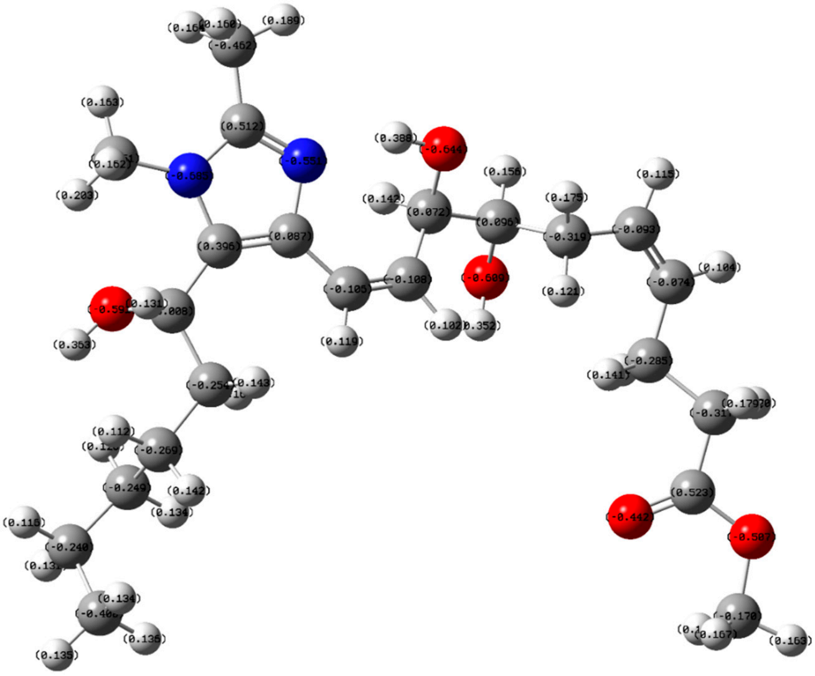

Figure S13: Mulliken charge distribution for analogue 2
